# Mitochondrial pathogenic mechanism and degradation in optineurin E50K mutation-mediated retinal ganglion cell degeneration

**DOI:** 10.1038/srep33830

**Published:** 2016-09-22

**Authors:** Myoung Sup Shim, Yuji Takihara, Keun-Young Kim, Takeshi Iwata, Beatrice Y. J. T. Yue, Masaru Inatani, Robert N. Weinreb, Guy A. Perkins, Won-Kyu Ju

**Affiliations:** 1 Hamilton Glaucoma Center, Department of Ophthalmology, and Shiley Eye Institute, University of California San Diego, La Jolla, CA, USA; 2 Department of Ophthalmology, Faculty of Medical Science, University of Fukui, Fukui, Japan; 3 Center for Research in Biological Systems, National Center for Microscopy and Imaging Research and Department of Neuroscience, University of California San Diego, La Jolla, CA, USA; 4 Division of Molecular and Cellular Biology, National Institute of Sensory Organs, National Hospital Organization Tokyo Medical Center, Tokyo, Japan; 5 Department of Ophthalmology and Visual Sciences, College of Medicine, University of Illinois at Chicago, IL USA

## Abstract

Mutations in optineurin (OPTN) are linked to the pathology of primary open angle glaucoma (POAG) and amyotrophic lateral sclerosis. Emerging evidence indicates that OPTN mutation is involved in accumulation of damaged mitochondria and defective mitophagy. Nevertheless, the role played by an OPTN E50K mutation in the pathogenic mitochondrial mechanism that underlies retinal ganglion cell (RGC) degeneration in POAG remains unknown. We show here that E50K expression induces mitochondrial fission-mediated mitochondrial degradation and mitophagy in the axons of the glial lamina of aged E50K^−tg^ mice *in vivo*. While E50K activates the Bax pathway and oxidative stress, and triggers dynamics alteration-mediated mitochondrial degradation and mitophagy in RGC somas *in vitro*, it does not affect transport dynamics and fission of mitochondria in RGC axons *in vitro*. These results strongly suggest that E50K is associated with mitochondrial dysfunction in RGC degeneration in synergy with environmental factors such as aging and/or oxidative stress.

Primary open angle glaucoma (POAG) is characterized by a slow and progressive degeneration of retinal ganglion cells (RGCs) and their axons that lead to loss of visual function^1^. It has been estimated that glaucoma will affect more than 80 million individuals worldwide by 2020 2. Despite the high prevalence, the biological basis of POAG still is not yet fully understood. Moreover, the genetic and environmental factors contributing to its progression are currently not well characterized. In this regard, mutations in the optineurin (*OPTN*) gene are associated with both normal tension glaucoma (NTG), a subset of adult-onset POAG, and amyotrophic lateral sclerosis (ALS)^3^,^4^, and may have a role in glaucomatous neurodegeneration.

Among the various mutations of *OPTN*, the E50K is the most prevalent, and it is associated with NTG^4^. OPTN, a highly expressed protein in RGCs^5^,^6^, has ubiquitous effects. In particular, it has been shown to be involved in the maintenance of Golgi organization^7^,^8^, regulation of nuclear factor kappa B (NF-κB) signaling^9^,^10^,^11^ and induction of autophagy and/or mitophagy^12^,^13^,^14^. Recent studies, including those from our laboratory, have demonstrated that E50K mutation triggers age-related RGC loss^15^, resulting in axonal degeneration and functional visual impairment in transgenic mice^16^. The precise pathophysiological mechanisms underlying the E50K mutation-mediated glaucomatous RGC degeneration however remain unclear.

Impairment of mitochondrial dynamics (fusion and fission) is thought to be critical in mitochondrial dysfunction in various neurodegenerative diseases including glaucoma^17^,^18^,^19^. Mitochondrial dynamics within healthy cells is regulated by a family of dynamin-related GTPases that exert opposing effects. While optic atrophy type 1 (OPA1) and mitofusins are required for mitochondria fusion, dynamin-related protein-1 (DRP1) regulates mitochondrial fission^20^,^21^,^22^. Our previous studies have demonstrated that glaucomatous insults impair mitochondrial dynamics and bioenergetics, and induce mitochondrial dysfunction, leading to RGC and its axon degeneration in rodent models of glaucoma^18^,^19^,^23^,^24^,^25^. More recent data further indicated that either OPA1 overexpression or DRP1 inhibition can enhance RGC survival and rescue its axon by preserving mitochondrial integrity in glaucomatous DBA/2J mice^19^,^23^. These findings strongly suggest the presence of a distinct mitochondrial dynamics-associated degenerative pathway in glaucomatous neurodegeneration.

In addition to the other effects, evidence has accumulated showing that OPTN also has a key role as an autophagy receptor in recruiting autophagy machinery^12^,^13^,^14^,^26^. It also contributes to the initiation of mitophagosome formation for the clearance of damaged mitochondria^13^,^14^. Of interest, an ALS-associated mutation in OPTN’s ubiquitin binding domain E478G is reported to cause defective mitophagy and accumulation of damaged mitochondria^13^. However, whether a mitochondrial pathogenic mechanism that includes mitochondrial dysfunction, degradation, and altered biogenesis also underlies E50K mutation-mediated RGC and axon degeneration in POAG/NTG has yet to be explored.

We report here for the first time that E50K mutation modifies the Bax pathway and alters mitochondrial dynamics, as well as triggers mitochondrial degradation and autophagosome and/or mitophagosome formation in aged E50K-mutation-carrying transgenic (E50K^−tg^) mice *in vivo* and primary RGCs *in vitro*.

## Results

### Overexpressed E50K mutation increases Bax protein expression, alters the OXPHOS complex (Cx) system and triggers oxidative stress in the retina of aged E50K^−tg^ mice

**Figure 1:**
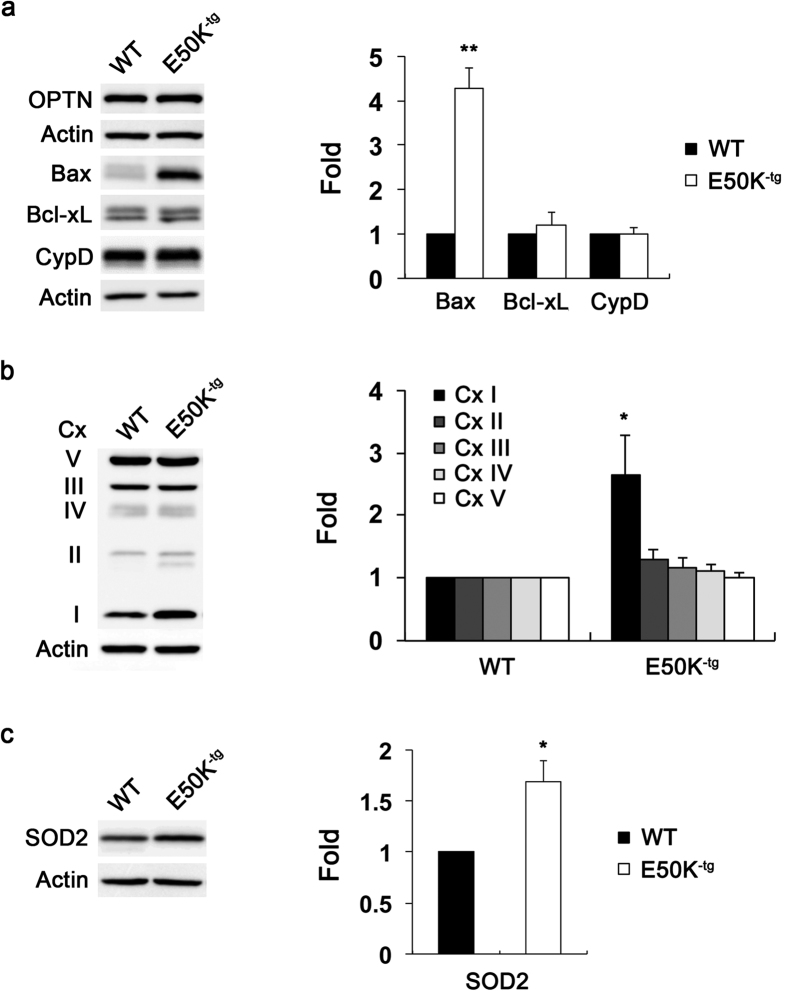
Increase of Bax and OXPHOS Cx expression in the retina of aged E50K^−tg^ mice. (**a**) Immunoblot analysis of Bax, Bcl-xL, CypD and OPTN protein in retinal extracts from 16-month-old aged E50K^−tg^ mice. (**b**) Immunoblot analysis of OXPHOS Cx protein in retinal extracts from 16-month-old E50K^−tg^ mice. (**c**) Immunoblot analysis of SOD2 protein expression in retinal extracts from 16-month-old E50K^−tg^ mice. For each determination, the actin level in age-matched WT mice was normalized to a value of 1.0. Data are shown as the mean ± S.D. (*n* = 3 independent experiments). *P < 0.05; **P < 0.01 compared with the WT group. Full-length blots are presented in Supplementary Figure 5. CypD, cyclophilin D; Cx, complex; E50K^−tg^, E50K mutation-carrying transgenic; OPTN, optineurin; SOD2, superoxide dismutase 2; WT, wild-type.

E50K^−tg^ or bacterial artificial chromosome-human OPTN^E50K^ mice displayed pathological phenotypes, such as age-related loss of RGCs and their axons, massive apoptosis or persistent reactive gliosis in the retina^15^,^16^. Using aged (16-month-old) E50K^−tg^ mice, we found that the E50K expression significantly increased Bax protein expression by 4.28 ± 0.46-fold in their retina compared with age-matched wild-type (WT) control mice (Fig. 1a). No statistically significant differences were detected, however, between E50K transgenic and control groups in the expression levels of Bcl-xL and cyclophilin D (CypD) proteins, which have roles in mitochondrial permeability transition pore (MPTP) opening-mediated apoptosis^27^,^28^ (Fig. 1a). We noted that the E50K mutation significantly increased the protein expression levels of mitochondrial OXPHOS complex I (Cx I) by 2.64 ± 0.63-fold in the retina of E50K^−tg^ mice compared with WT mice, respectively (Fig. 1b). Additionally, we found that the E50K mutation significantly enhanced the protein expression levels of superoxide dismutase 2 (SOD2) by 1.69 ± 0.20-fold in the retina of E50K^−tg^ mice compared with WT mice, respectively (Fig. 1c).

Consistent with our previous finding of RGC loss^15^, E50K significantly decreased in E50K^−tg^ mice the expression level of brain-derived neurotrophic factor (BDNF) protein, a major pro-survival factor that enhances RGC survival^29^, by 80% (from 1.0 to 0.20 ± 0.08) in the retina (Supplementary Figure 1a). BDNF can be regulated by cyclic AMP response element-binding protein (CREB)^30^. It is also known to initiate CREB phosphorylation in the rat optic nerve^31^. We therefore determined whether E50K mutation causes BDNF loss by altering CREB phosphorylation at serine 133 (S133) in cultured RGCs. The results showed that there was no statistically significant difference in the expression level of phosphorylated CREB S133 (Supplementary Figure 1a), suggesting that the BDNF loss might be related to RGC axon degeneration rather than CREB phosphorylation in the RGC soma. Immunohistochemical analysis further revealed that the E50K mutation induced not only a significant loss of BDNF immunoreactivity in RGCs of the ganglion cell layer (GCL) but also an increase of glial fibrillary acidic protein (GFAP) expression in müller cells of the inner retinal layer in the peripheral retina of aged E50K^−tg^ mice compared with WT mice (Supplementary Figure 1b).

### Overexpression of E50K results in fission-mediated mitochondrial degradation and mitophagy in the axons of the glial lamina in aged E50K^−tg^ mice

**Figure 2:**
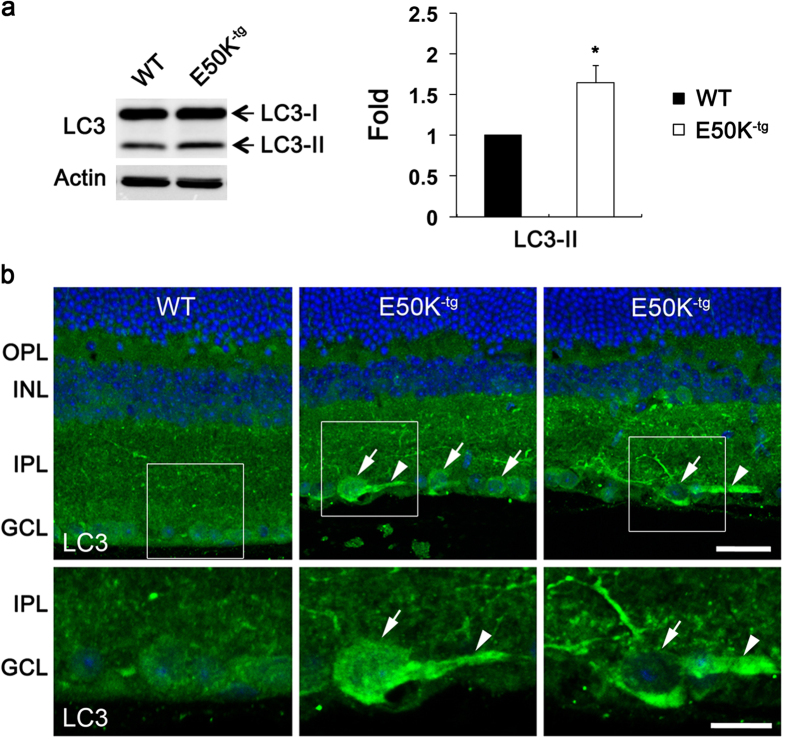
Increase of LC3 protein expression in RGCs and their axons of aged E50K^−tg^ mice. (**a**) Immunoblot analysis of LC3 protein in retinal extracts from 16-month-old aged E50K^−tg^ mice. For each determination, the actin level in age-matched WT mice was normalized to a value of 1.0. Data are shown as the mean ± S.D. (*n* = 3 independent experiments). *P < 0.05 compared with the WT group. Full-length blots are presented in Supplementary Figure 6. (**b**) Immunohistochemical analysis of LC3 protein expression in retinal sections from WT and E50K^−tg^ mice. Both RGC somas and axons increased LC3 immunoreactivity in E50K^−tg^ mice. Arrows indicate LC3-positive RGC somas in the GCL and arrowheads indicate LC3-positive RGC axons in the GCL. E50K^−tg^, E50K mutation-carrying transgenic; GCL, ganglion cell layer; INL, inner nuclear layer; IPL, inner plexiform layer; LC3, microtubule-associated protein 1A/1B-light chain 3; OPL, outer plexiform layer; OPTN, optineurin; RGC, retinal ganglion cell; WT, wild-type. Scale bar, 20 μm.

OPTN has been shown to play a role as an autophagy receptor via binding to LC3 to clear damaged mitochondria by initiating mitophagosome formation^13^,^14^. We first determined whether the E50K mutation increased autophagy in RGCs of aged E50K^−tg^ mice. Indeed, E50K expression significantly enhanced LC3-II protein level by 1.65 ± 0.20-fold in the retina of aged E50K^−tg^ mice compared with WT mice (Fig. 2a). By immunohistochemical analysis, a strong LC3 immunoreactivity in the RGC somas and their axons was observed in the GCL of aged E50K^−tg^ mice (Fig. 2b).

**Figure 3:**
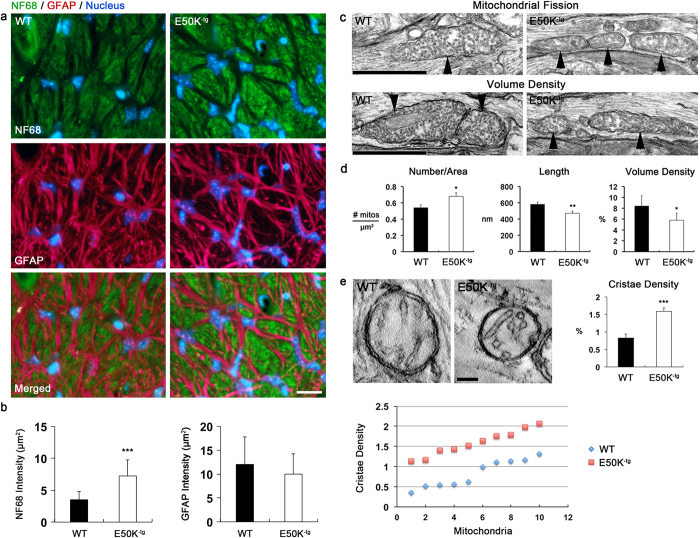
Induction of fission-mediated mitochondrial loss in the axons of the glial lamina in aged E50K^−tg^ mice. (**a**,**b**) Representative images and quantitative immunoreactive intensity analyses from the glial lamina show a significant increase of NF68 immunoreactivity (green) in the axons of 16-month-old aged E50K^−tg^ mice compared with age-matched WT mice. There were no differences in astroglial activation and GFAP immunoreactivity (red) between WT and E50K^−tg^ mice. Blue color indicates nucleus. Scale bar, 20 μm (**c**) Representative images from TEM analyses show mitochondrial fission and mitochondrial loss in the axons of the glial lamina in 16-month-old aged E50K^−tg^ mice compared with age-matched WT mice. Arrowheads indicate mitochondria. Scale bar, 500 nm. (**d**) Quantitative TEM analyses of mitochondria show a significant increase of mitochondrial number (*n* = 20), but decreases of mitochondrial lengths (*n* = 78 for WT; 92 for E50K^−tg^ mice) and volume density (*n* = 20) in the axons of the glia lamina of E50K^−tg^ mice. (**e**) Representative images from TEM analyses show changes in cristae abundance in the mitochondria of the axons in the glial lamina in WT and E50K^−tg^ mice. Quantitative TEM analysis shows a significant increase of mitochondrial cristae density (*n* = 10 for WT; 10 for E50K^−tg^ mice) in the axons of the glial lamina in E50K^−tg^ mice. Dot graph shows the actual cristae density of each mitochondrion. Data are shown as the mean ± S.E.M. *P < 0.05; **P < 0.01; ***P < 0.001 compared with the WT group. E50K^−tg^, E50K mutation-carrying transgenic; GFAP, glial fibrillary acidic protein; **NF68, neurofilament 68**; WT, wild-type. Scale bar, (WT) 500 nm (c), (E50K^−tg^) 250 nm (**c**) and (WT and E50K^−tg^) 100 nm (**e**).

We next assessed whether the E50K expression is associated with alteration of mitochondrial dynamics by measuring mitochondrial number per area, membrane surface areas and volume density, as well as formation of autophagosomes and/or mitophagosomes in RGC axons of the glial lamina in aged E50K^−tg^ mice. Immunohistochemical analysis showed that the E50K mutant triggered abnormal accumulation of axonal contents with a significantly increased neurofilament 68 (NF68) immunoreactivity by 7.23 ± 2.50 per μm^2^ in the glial lamina of aged E50K^−tg^ mice compared with WT mice (3.47 ± 1.32 per μm^2^) (Fig. 3a,b). However, there were no statistical differences in GFAP immunoreactivity between aged E50K^−tg^ and WT mice (Fig. 3a,b). Of interest, representative images from transmission electron microscopy (TEM) analyses presented fragmented and generally smaller mitochondria in the axons of the glial lamina in E50K^−tg^ mice (Fig. 3c). Quantitative TEM analyses additionally disclosed a significant increase of mitochondrial number (0.68 ± 0.05 per μm^2^), but decreases of mitochondrial lengths (0.47 ± 0.03 μm) and volume density (5.8 ± 1.3%) in the axons of the glial lamina of aged E50K^−tg^ mice compared with WT mice (0.54 ± 0.04 per μm^2^, 0.58 ± 0.03 μm, and 8.5 ± 1.9%, respectively) (Fig. 3d). In addition, representative images from TEM analysis presented cristae structures in the mitochondria of the axons in the glial lamina in E50K^−tg^ mice (Fig. 3e). Quantitative TEM analyses showed a significant increase of cristae density (1.58 ± 0.1%) in the mitochondria of the axons in the glial lamina of aged E50K^−tg^ mice compared with WT mice (0.82 ± 0.11%) (Fig. 3e).

**Figure 4:**
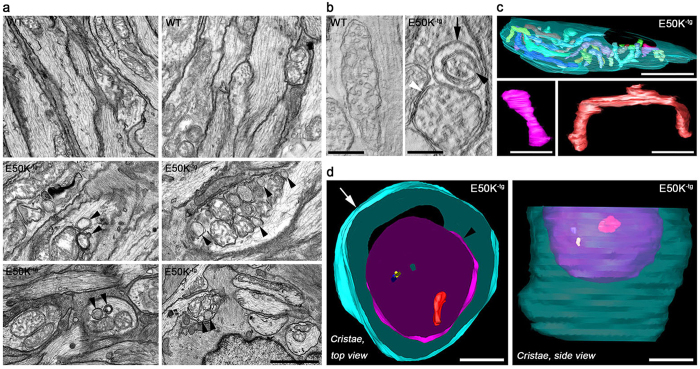
Formation of autophagosome and mitophagosome in the axons of the glial lamina in aged E50K^−tg^ mice. (**a**) Representative images from TEM analyses show several examples of autophagosome formation in the axons of the glial lamina in 16-month-old aged E50K^−tg^ mice compared with age-matched WT mice. Arrowheads indicate formation of autophagosomes in several examples of evulsions showing an increased number of degrading vacuoles and spherical structures with double layer membranes. Scale bar, 500 nm. (**b**) Electron tomography generated high-resolution 3D reconstructions of mitochondria and mitophagosome formation in the axons of the glial lamina of WT and E50K^−tg^ mice. An example of a degraded small mitochondrion (black arrowhead) engulfed in a mitophagosome (black arrow) is seen in E50K^−tg^ mice. The white arrowhead points to an adjacent mitochondrion that appears abnormal and has been separated from its neighboring larger mitochondrion (not shown) with normal appearance. (**c**) Surface-rendered views of WT volume segmentation show a normal mitochondrion (transparent cyan outer membrane; cristae of various colors) with numerous well-formed cristae. Typically, the cristae were either tubular (lower left, pink) or branched (lower right, red). (**d**) An example of a mitophagosome in the axon of the glial lamina in aged E50K^−tg^ mice. Surface-rendered views of volume segmentation show a degraded small mitochondrion (black arrowhead, magenta) engulfed in the mitophagosome (arrow, cyan). The damaged mitochondrion exhibits severe cristae depletion (only 3 very small cristae shown in red, blue and yellow) as seen in the top and side views. The perspective of the volumes occupied by the mitochondrion (purple) and its 3 small cristae inside the mitophagosome is best seen from a side view (right). E50K^−tg^, E50K mutation-carrying transgenic; WT, wild-type. Scale bar, (WT) 500 nm, (E50K^−tg^) 250 nm.

Furthermore, the E50K expression induced autophagosome formation. Using TEM, an increased number of degrading vacuoles and spherical structures with double layer membranes were seen in the axons of the glial lamina in E50K^−tg^ mice (Fig. 4a). Notably, mitophagosome formation was also observed (Fig. 4b). In comparison with axonal mitochondria that were filled with tubular and branched cristae in WT mice (Fig. 4c), segmented volumes from three-dimensional (3D) tomographic reconstructions revealed degraded mitochondria with severe cristae depletion engulfed in mitophagosomes in the glial lamina of aged E50K^−tg^ mice (Fig. 4d).

### Overexpression of E50K increases Bax protein levels in RGCs *in vitro*

**Figure 5:**
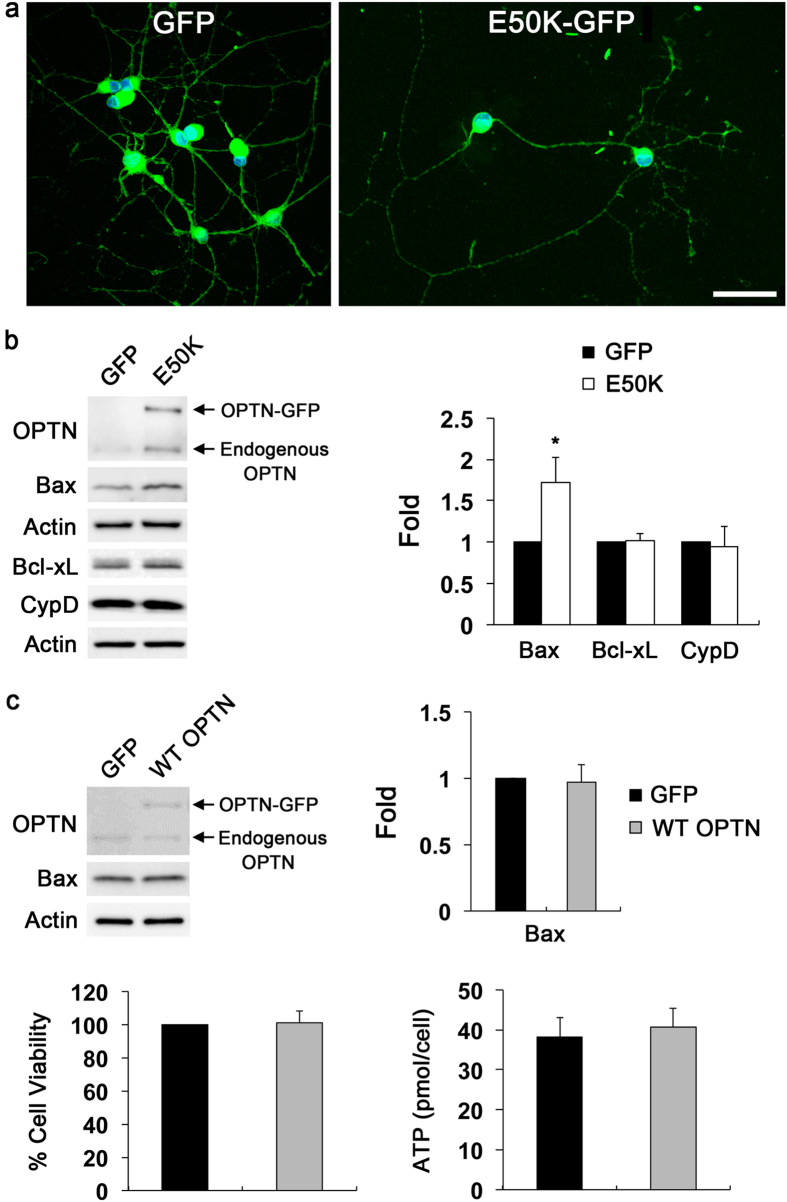
Overexpression of E50K promotes Bax protein expression in RGCs *in vitro*. (**a**) Primary RGCs were transduced with AAV2-GFP, AAV2-OPTN_WT_-GFP or AAV2-OPTN_E50K_-GFP for 2 days *in vitro*. Representative images show GFP-positive primary RGCs after transduction. Scale bar, 2 μm. (**b**) Immunoblot analyses of OPTN, Bax, Bcl-xL and CypD protein in cultured RGCs overexpressing E50K mutant. For each determination, the actin level in control was normalized to a value of 1.0. (**b**) Immunoblot analyses of OPTN, Bax, Bcl-xL and CypD protein in cultured RGCs overexpressing WT OPTN. For each determination, the actin level in control was normalized to a value of 1.0. Data are shown as the mean ± S.D. (*n *= 3 independent experiments). *P < 0.05 compared with the control group. Full-length blots are presented in Supplementary Figure 7. CypD, cyclophilin D; GFP, green fluorescent protein; OPTN, optineurin; RGC, retinal ganglion cell; WT, wild-type.

We determined herein whether E50K mutation alters the expression level of Bax protein in cultured RGCs. Purified RGCs were transduced with adeno-associated virus serotype 2 (AAV2)-green fluorescent protein (GFP) or AAV2-OPTN_E50K_-GFP for 2 days. In addition, we also transduced AAV2-OPTN_WT_-GFP as a control to determine the specificity of the consequence of the E50K mutation in cultured RGCs. Following confirmation of GFP and E50K-GFP expression in cultured RGCs after transduction (Fig. 5a), the E50K expression in RGCs was found to increase Bax protein level by 1.72 ± 0.3-fold (*P* < 0.05) compared with control cells (Fig. 5b), correlating with our findings from retina of aged E50K^−tg^ mice (Fig. 1a). By contrast, there were no statistically significant differences in the expression levels of Bcl-xL and CypD between E50K and control groups (Fig. 5b). Again, in agreement with our findings from retina of aged E50K^−tg^ mice (Supplementary Figure 1), we found that E50K expression triggered a significant loss of BDNF protein by 0.72 ± 0.05-fold in cultured RGCs but it did not affect the CREB S133 phosphorylation level (Supplementary Figure 2).

Additionally, immunoblotting experiments demonstrated that there was no statistically significant difference in the expression level of Bax protein between WT OPTN and control groups (Fig. 5c), suggesting that the increased Bax protein expression observed was specific to E50K mutation. Moreover, there were no statistically significant differences in cell viability and cellular ATP production by a 3-[4,5-dimethylthiazol-2yl]-2,5-diphenyl tetrazolium bromide (MTT) assay and luciferase-based assay, respectively, between WT OPTN and control groups (Fig. 5c).

### Overexpression of E50K alters OXPHOS Cx system and mitochondrial dynamics in RGCs *in vitro*

**Figure 6:**
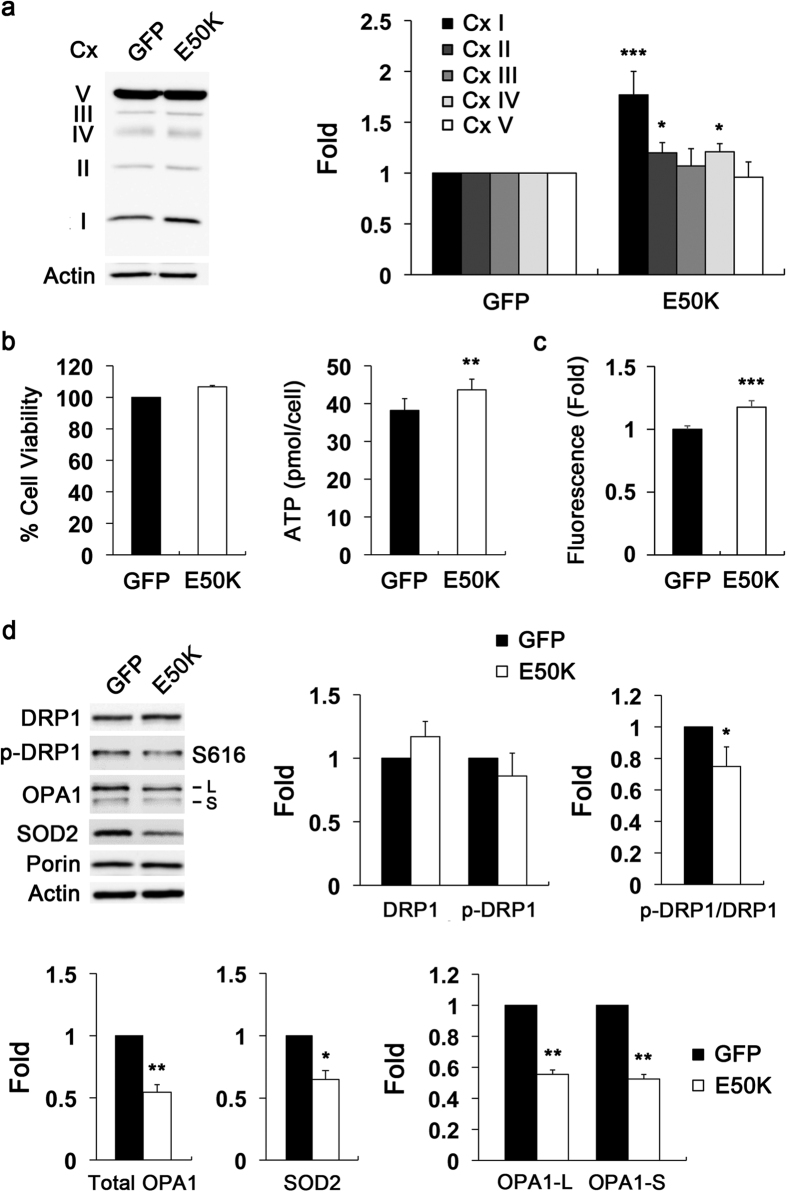
Overexpression of E50K alters OXPHOS Cx system, increases ROS production, and triggers mitochondrial fission and OPA1 loss in RGCs *in vitro*. Primary RGCs were transduced with AAV2-GFP or AAV2-OPTN_E50K_-GFP for 2 days *in vitro*. (**a**) Immunoblot analysis of OXPHOS Cx protein in cultured RGCs overexpressing GFP (control) or E50K mutant. (**b**) Analyses of cell viability using the MTT assay and cellular ATP production using a luciferase-based assay in cultured RGCs overexpressing GFP (control) or E50K mutant. (**c**) ROS measurement in cultured RGCs overexpressing GFP (control) or E50K mutant. (**d**) Immunoblot analyses of DRP1, phospho-DRP1 (S616), OPA1, SOD2 and porin protein in cultured RGCs overexpressing GFP (control) or E50K mutant. For each determination, the actin level in the control was normalized to a value of 1.0. Data are shown as the mean ± S.D. (*n *= 3 independent experiments). *P < 0.05; **P < 0.01; ***P < 0.001 compared with the control group. Full-length blots are presented in Supplementary Figure 8. CypD, cyclophilin D; Cx, complex; DRP1, dynamin-related protein 1; GFP, green fluorescent protein; L, large form; S, small form; OPA1, optic atrophy type 1; OPTN, optineurin; RGC, retinal ganglion cell; ROS, reactive oxygen species; S616, serine 616; SOD2, superoxide dismutase 2.

Based on our findings of increase of OXPHOS Cx I and Bax protein expression in the retina of aged E50K^−tg^ mice and cultured E50K-expressing RGCs (Figs 1 and 5), as well as a previous report of Bax-mediated impairment of mitochondrial respiration^32^, we determined further whether E50K expression would alter OXPHOS Cx system in cultured RGCs. Purified RGCs were transduced with AAV2-GFP or AAV2-OPTN_E50K_-GFP for 2 days. By Western blotting, E50K was noted to significantly increase the protein levels of OXPHOS Cxs (Cx I, II and IV) by 1.69 ± 0.08-, 1.20 ± 0.01- and 1.22 ± 0.01-fold respectively in cultured RGCs compared with controls (Fig. 6a). However, E50K did not induce statistically significant differences in the expression levels of other OXPHOS Cxs (Cx III and V). While the cell viability was not found statistically changed by MTT assay, the E50K expression did cause a significant increase in cellular ATP production (43.6 ± 2.83 pmol/cell) in cultured RGCs compared with control cells (38.2 ± 3.14 pmol/cell) (Fig. 6b). Since recent studies reported that the impairment of OXPHOS is associated with alteration of mitochondrial dynamics and reactive oxygen species (ROS) production^33^,^34^, we examined in addition whether E50K expression alters ROS production and mitochondrial dynamics-related proteins, OPA1 and DRP1, and SOD2 protein in cultured RGCs. We found that E50K significantly increased intracellular ROS production by 1.18 ± 0.05-fold in cultured RGCs compared with control cells (Fig. 6c). Surprisingly, E50K significantly decreased the expression levels of DRP1 S616 phosphorylation (normalized to total DRP1) by 0.74 ± 0.15-fold, OPA1 [both large (100 kDa) and small (80 kDa) forms] by 0.78 ± 0.09- and 0.77 ± 0.09-fold respectively, as well as SOD2 by 0.66 ± 0.07-fold in cultured RGCs compared with control cells (Fig. 6d).

### Overexpression of E50K causes mitochondrial loss and mitophagy in RGC somas *in vitro*

**Figure 7:**
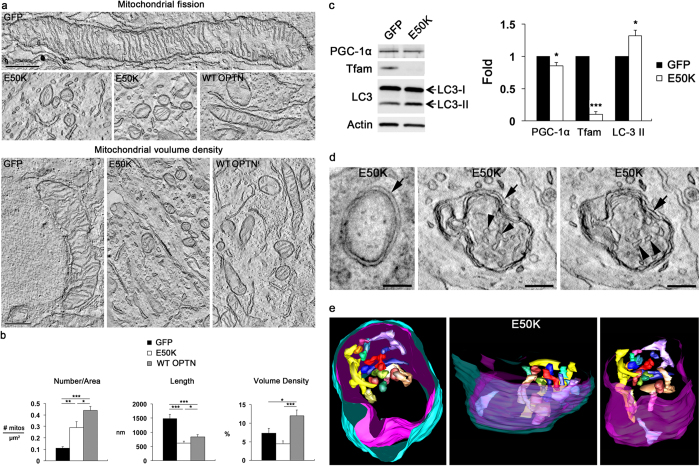
Overexpression of E50K triggers fission-associated mitochondrial loss and autophagosome and mitophagosome formation in RGC somas *in vitro*. Primary RGCs were transduced with AAV2-GFP, AAV2-OPTN_E50K_-GFP or AAV2-OPTN_wt_-GFP for 2 days *in vitro*. (**a**) Representative images from electron tomography analyses show large-scale fissioning of mitochondria and alteration of mitochondrial volume density in cultured RGC somas overexpressing E50K mutant or WT OPTN. Scale bar, 500 nm. (**b**) Quantitative electron tomography analyses of mitochondrial number (*n *= 10), mitochondrial lengths (*n *= 50) and mitochondrial volume density (*n *= 10) in cultured RGC somas overexpressing E50K mutant or WT OPTN. Data are shown as the mean ± S.E.M. **P < 0.01; ***P < 0.001 compared with the GFP control group. (**c**) Immunoblot analyses of SOD2, PGC-1, Tfam and LC3 protein in cultured RGCs overexpressing E50K mutant. For each determination, the actin level in control was normalized to a value of 1.0. Data are shown as the mean ± S.D. (*n *= 3 independent experiments). *P < 0.05; ***P < 0.001 compared with the control group. Full-length blots are presented in Supplementary Figure 9. (d) Electron tomography generated high-resolution reconstructions of an autophagosome (left, arrow) and a mitophagosome (middle and right, arrows) in cultured RGC somas overexpressing E50K mutant. Arrowheads point to representative cristae. (**e**) Surface-rendered views of volume segmentation of the mitophagosome shown in panel (**d**) The mitochondrion (magenta) engulfed in the mitophagosome (cyan) occupies most of the volume of the mitophagosome as seen in this top view (left). The entire complement of cristae found inside the mitochondrion is shown in various colors. All but one of the cristae are aggregated towards one side of the mitochondrion shown in this side view (middle), indicating polarized damage to this mitochondrion. The mitophagosome and mitochondrial outer membranes were made transparent to better see the cristae. Another view with the mitophagosome membrane not shown (right). Only the mitochondrial outer membrane and cristae are seen in this oblique view. GFP, green fluorescent protein; LC3, microtubule-associated protein 1A/1B-light chain 3; OPTN, optineurin; PGC-1α, peroxisome proliferator-activated receptor-γ coactivator (PGC)-1 alpha; RGC, retinal ganglion cell; SOD2, superoxide dismutase 2; Tfam, mitochondrial transcription factor A.

We subsequently examined the alteration of the mitochondrial network by measuring mitochondrial number per area, membrane surface areas and volume density, as well as formation of autophagosomes and/or mitophagosomes in cultured RGC somas. Purified RGCs were transduced with AAV2-GFP or AAV2-OPTN_E50K_-GFP for 2 days. Again, we also transduced AAV2-OPTN_WT_-GFP as a positive control to determine the specificity of the consequence of the E50K mutation in cultured RGCs. Representative images from electron tomography analyses revealed that E50K-overexpressing cells contained fragmented and consistently smaller mitochondria. Reduction of mitochondrial volume density in the somas of E50K- RGCs was also noted in comparison with control cells that most often contained a tubular form of elongated mitochondria (Fig. 7a). Quantitative electron tomography analyses indicated that E50K triggered a significant increase of mitochondrial number (0.29 ± 0.05 per μm^2^), but decreases of mitochondrial lengths (0.62 ± 0.07 μm) and volume density (4.50 ± 0.74%) in cultured RGC somas compared with control cells expressing GFP alone (0.11 ± 0.01 per μm^2^, mitochondrial number; 1.48 ± 0.15 μm, mitochondrial lengths; and 7.30 ± 0.13%, volume density) (Fig. 7b).

On the other hand, representative images from electron tomography analyses showed that WT OPTN expression yielded changes to mitochondria in cultured RGC somas (Fig. 7a). Surprisingly, quantitative electron tomography analyses revealed that WT OPTN led to a significant increase in mitochondrial number (0.44 ± 0.03 per μm^2^), but decrease in mitochondrial lengths (0.83 ± 0.08 μm) in the somas of cultured RGCs compared with control cells expressing GFP alone (Fig. 7b). Of interest, a significant increase in mitochondrial volume density (12.0 ± 1.5%) was observed in WT OPTN-expressing cultured RGCs compared with control cells expressing GFP alone (Fig. 7b), suggesting that WT OPTN may induce mitochondrial biogenesis in cultured RGC somas.

E50K expression also significantly decreased the expression levels of peroxisome proliferator-activated receptor-gamma coactivator (PGC)-1α and mitochondrial transcription factor A (Tfam) protein by 0.85 ± 0.05- and 0.1 ± 0.04-fold respectively in E50K-expressing RGCs compared with GFP controls (Fig. 7c). Furthermore, ectopically expressed E50K significantly increased LC3-II protein expression by 1.32 ± 0.08-fold in cultured RGCs (Fig. 7c). In agreement with this finding, representative images from electron tomography analyses displayed not only formation of autophagosomes that had the defining double autophagosome membrane, but also mitophagosomes that contained well-defined cristae in the degenerating mitochondria enveloped in this type of autophagosomes in cultured RGC soma (Fig. 7d). Often, that the mitochondrion engulfed in the mitophagosome occupied most of the volume of the mitophagosome as seen from a top view (Fig. 7e). The entire complement of cristae found inside the mitochondrion is shown in various colors in Fig. 7e. All but one of the cristae were aggregated towards one side of the mitochondrion (side view, Fig. 7e), implying that polarized damage had occurred to this mitochondrion (Fig. 7e; Supplementary Movie 1). Collectively, these results suggested that E50K expression triggered mitochondrial degradation and mitophagy in RGC somas *in vitro*.

### Overexpression of E50K did not alter the transport dynamics and length of transported mitochondria in RGC axons *in vitro*

**Figure 8:**
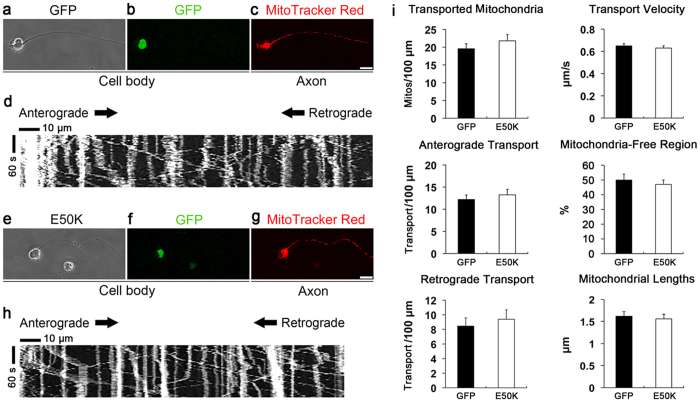
Overexpression of E50K did not alter transport dynamics and lengths of transported mitochondria in cultured RGC axons. Primary RGCs were transduced with AAV2-GFP or AAV2-OPTN_E50K_-GFP for 2 days *in vitro*. *In vitro* time-lapse imaging shows no significant effects of OPTN E50K mutation on the transport dynamics and lengths of transported mitochondria in the axons of cultured RGCs. (**a**,**b**) Representative phase-contrast (**a**) and fluorescence (**b**, GFP) images of an RGC with AAV2-GFP focused on the cell body. (**c**) A representative fluorescence (MitoTracker Red) image of the RGC with AAV-GFP focused on the axon (Supplementary Movie 2). (**d**) A kymograph (a representation of mitochondrial positions in an axon during the recording time) of the axon in c detected active axonal transport of mitochondria shown as diagonal lines. (**e**,**f**) Representative phase-contrast (**e**) and fluorescence (**f**, GFP) images of an RGC with AAV2-OPTN_E50K_-GFP focused on the cell body. (**g**) A representative fluorescence (MitoTracker Red) image of the RGC with AAV2-OPTN_E50K_-GFP focused on the axon (Supplementary Movie 3). Scale bar, 20 μm. (**h**) A kymograph of the axon in (**g**) detected active axonal transport of mitochondria. (**i**) No significant effects of the E50K expression were observed in either the number of mitochondria transported in axons (*P* = 0.36), the anterograde (*P* = 0.55) and retrograde (*P* = 0.61) transport, transport velocity (*P* = 0.32), mitochondria-free regions (*P* = 0.66), or lengths of transported mitochondria (*P* = 0.29) in axons. Data are shown as the mean ± S.E.M. GFP, *n* = 261 mitochondria from 8 axons; and E50K, *n* = 302 mitochondria from 10 axons. (*n* = 3 independent experiments). GFP, green fluorescent protein; OPTN, optineurin; RGC, retinal ganglion cell.

Since the present study revealed fission-mediated mitochondrial loss in RGC axons of the glial lamina of E50K^−tg^ mice, we further determined whether the overexpressed E50K alters the transport dynamics and lengths of axonal mitochondria in cultured RGCs. Purified RGCs were transduced with AAV2-GFP or AAV2-OPTN_E50K_-GFP for 2 days. *In vitro* time-lapse live imaging (every 3 s for 3 min) of axonal transport of mitochondria was performed on RGCs, which had extended axons (Fig. 8a,c,e,g) and were positive for GFP (Fig. 8b,f). MitoTracker Red CMXRos (12.5 nM) was used to visualize mitochondria in GFP-positive RGC axons (Fig. 8,b,c,f,g). Active axonal transport of mitochondria was seen in both the RGCs transduced with AAV2-GFP (Supplementary Movie 2) and AAV2-OPTN_E50K_-GFP (Supplementary Movie 3). Kymographs (representation of mitochondrial positions in an axon during the recording time) of the axons with AAV2-GFP (Fig. 8d) and AAV2-OPTN_E50K_-GFP (Fig. 8h) demonstrated active axonal transport dynamics of mitochondria in the anterograde and retrograde directions shown as diagonal lines. Surprisingly, quantitative analyses using kymographs detected no statistically significant differences between GFP and E50K groups on the number of transported mitochondria, anterograde and retrograde transport, transport velocity, mitochondria-free regions, and lengths of transported mitochondria in the axons of cultured RGCs (Fig. 8i). The averages of transport velocity in each of the two groups converged toward approximately 0.6 μm/s (Fig. 8i).

## Discussion

Several susceptible genes and environmental factors have been highlighted as potential therapeutic targets for POAG, the most common form of glaucoma^35^,^36^. OPTN has been a focus of research since mutations in OPTN are linked to the pathology of NTG, a subtype of POAG, and ALS^3^,^4^. Emerging evidence demonstrates that OPTN acts as an autophagy receptor, and contribute to mitochondrial degradation via mitophagy^13^,^14^. Among various OPTN mutations in NTG patients, the OPTN E50K mutation has been associated with a rapidly progressive course and severe disease. Age-related loss of RGC and its axons by E50K overexpression is also seen in experimental mouse models^15^,^16^. It is notable that the E50K mutation-mediated molecular events and their connection to RGC and axonal degeneration are still poorly understood. To determine the significance of a putative pathogenic mitochondrial pathway and its synergistic effect with aging and/or oxidative stress in E50K mutation-induced RGC degeneration, the present study utilized both an *in vivo* E50K^−tg^ mouse model and *in vitro* primary RGC cultures overexpressing E50K using an AAV transduction system.

In the current study, Bax protein expression was found to be enhanced in RGCs overexpressing E50K *in vitro*. In aged E50K^−tg^ mice, a significant increase of Bax protein expression was also noted in retinal extracts. Bax has been shown previously to contribute to RGC susceptibility in glaucomatous neurodegeneration^18^,^37^,^38^. Collectively, these findings suggest that an increased Bax activity could be associated with the signaling mechanism mediated by E50K to induce RGC degeneration. Bax-mediated mitochondrial outer membrane permeabilization (MOMP) is an important pathophysiological mechanism for OXPHOS alteration, ROS production or apoptosis^39^,^40^. Loss of OXPHOS Cx I activity increases ROS levels and induces apoptosis^41^. OXPHOS Cx II is a source of the mitochondrial reserve respiratory capacity (RRC) that is regulated by metabolic sensor and promotes cell survival against hypoxia^42^. In the present study, while OXPHOS Cx I was increased *in vivo* in retinas of aged E50K^−tg^ mice, both OXPHOS Cx I and II were increased *in vitro* in E50K-overexpressing RGCs. In addition, intracellular ROS production was increased *in vitro* in E50K-overexpressing RGCs. These results suggest that E50K expression may alter mitochondrial respiratory chain function and ROS production in RGCs. Furthermore, because E50K overexpression did not affect the CypD protein level either in aged E50K^−tg^ mice or in cultured RGCs, it is possible that E50K may promote Bax-specific MOMP, and consequently lead to alterations of OXPHOS Cx I and/or II in RGCs and increased production of ROS in RGCs. Therefore, the current findings raise the possibility that Bax-associated alteration of OXPHOS Cxs I and II and their downstream ROS production and/or mitochondrial RRC may be involved in mitochondrial dysfunction-mediated RGC susceptibility.

Impairment of mitochondrial dynamics is associated with mitochondrial dysfunction in neurodegenerative diseases, including glaucoma^18^,^19^, ALS^43^ and central nervous system (CNS) aging^44^,^45^. Bax translocates to discrete foci on the mitochondrial membrane during the initial stage of apoptosis, which subsequently become mitochondrial scission sites and contribute to mitochondrial remodeling of the mitochondrial network^46^. Our recent studies demonstrated that glaucomatous damage increases *Bax* gene and protein expression in the retina, as well as renders fission-mediated mitochondrial loss by altering *OPA1* or *DRP1* gene and protein expression in RGC axons of the glial lamina in a mouse model of glaucoma, DBA/2J mice^18^,^19^,^37^. Emerging evidence from our group revealed that upon aging RGC axons increase mitochondria-free regions and decrease lengths of transported mitochondria^44^, suggesting that mitochondrial dynamics is altered with aging and may underlie the age-related increase in glaucoma incidence. Here we show that E50K expression alters mitochondrial dynamics and triggers mitochondrial degradation, and may thereby compromise mitochondrial dynamics in the axons of the glial lamina in aged E50K^−tg^ mice *in vivo* and in the somas of RGCs *in vitro*. Moreover, E50K is shown to induce significant decreases of OPA1 protein expression and DRP1 S616 phosphorylation in RGCs *in vitro*. These results represent the first direct evidence of E50K mutation-mediated impairment of mitochondrial dynamics in RGC degeneration.

We have previously reported that OPA1 deficiency induces increases of *Bax* gene and protein expression, as well as mitochondrial fission and an accompanying decrease of SOD2 protein expression, that may lead to oxidative stress and the initial stage of apoptosis in the retina of *Opa1*^enu*/*+^ mouse, a model of autosomal dominant optic atrophy^47^. Therefore, the decrease of SOD2 protein expression in E50K-expressing RGCs *in vitro* is likely related to the reduction of OPA1 protein expression. SOD2 deficiency has been shown to result in mitochondrial oxidative stress by increasing ROS production and contributes to retinal degeneration in a mouse model of genetic *Sod2* deficiency^48^. In contrast, it has been reported that SOD2 overexpression reduced E50K-induced cell death and oligomer formation, as well as ROS production^49^,^50^. In good agreement with these findings, the finding of reduced SOD2 protein expression was correlated with increased ROS production. In addition, we also note that E50K expression leads to a significant increase in cellular ATP production without altering cell viability in RGCs *in vitro*, suggesting that E50K expression may be associated with the early phase of apoptosis, which requires energy^51^, and increases cellular ATP production in viable RGCs. These findings collectively indicate that the overexpressed E50K may trigger oxidative stress by increasing ROS production and initiate the early stage of apoptosis via compromised mitochondrial dynamics and SOD2 deficiency in RGC degeneration.

In the current study, it is also shown that in RGCs *in vitro*, overexpressed E50K was found to induce significant decreases of expression of PGC-1α and Tfam, two proteins that play a critical role in mitochondrial biogenesis in mammalian cells^52^,^53^. Impaired mitochondrial biogenesis is an important mechanism in human diseases with mitochondrial defects and CNS aging. The decreased PGC-1α and Tfam protein expression suggest that E50K provokes the decline of mitochondrial biogenesis. Our results together suggest, therefore, that activation of the Bax pathway, impairment of mitochondrial dynamics, reduction of mitochondrial biogenesis, and a consequent degradation of mitochondria induced by E50K overexpression could be important mitochondrial pathogenic mechanisms in RGC degeneration of the POAG/NTG pathogenesis. Future studies will be needed to understand the relevance of these factors during glaucomatous neurodegeneration using *in vivo* animal models and an *in vitro* primary RGC culture system.

Of interest, it is demonstrated here that while overexpression of WT OPTN in RGCs *in vitro* did not change the expression level of Bax protein as well as cell viability and cellular ATP production, overexpression of WT OPTN triggered mitochondrial biogenesis, as supported by induced mitochondrial fission and increased mitochondrial volume density in RGCs *in vitro*. Because our group previously showed that enhanced WT OPTN upregulated toxic effects such as apoptosis *in vitro*^8^,^54^ as well as induced RGC and its axon degeneration in rats *in vivo*^55^, it implies that the induction of mitochondrial biogenesis could provide an important endogenous compensation mechanism in response to WT OPTN overexpression-mediated mitochondrial alteration in RGCs. Previously, OPTN deficiency has been associated with the vulnerability of striatal neurons in a mouse model of Huntington’s disease^56^ as well as the induction of apoptosis and reduction of neurotrophins secretion in RGC-5 cells (now recognized to be derived possibly from the 661W photoreceptor cell line^57^,^58^). Furthermore, it has been reported that OPTN promoted neuronal cell survival against glutamate-induced neurotoxicity, suggesting a possibility that OPTN is neuroprotective^6^,^59^,^60^. We therefore cannot exclude another possibility that increasing WT OPTN expression may have a beneficial effect for glaucomatous optic neuropathy and other neurodegenerative diseases. In the present study, we demonstrated a reduction of endogenous OPTN protein expression, accompanied by increased Bax protein expression, in retinal cells, including RGCs, of glaucomatous DBA/2J mice (Supplementary Figures 3 and 4). These findings suggest an intriguing possibility that the reduction of OPTN may contribute to neuronal cell death through altered mitochondrial dynamics or biogenesis, or via mitochondria-related degenerative pathways such as the Bax-mediated apoptotic pathway, in certain types of glaucomatous optic neuropathy. Future studies will be needed to clarify the role of WT OPTN in mitochondrial dynamics, biogenesis and dysfunction, as well as the apoptotic pathway in glaucomatous RGC degeneration.

Consistent with the previous demonstration that E50K triggers LC3-II-mediated induction of autophagy in the rat retina^55^ as well as induces autophagy-associated retinal cell death^61^,^62^, it is shown here that there was not only an increase of LC3-II protein expression in RGCs and their axons in the retina but also autophagosome and/or mitophagosome formation, accompanied by fission-mediated mitochondrial degradation, in the axons of the glial lamina in aged E50K^−tg^ mice. Surprisingly, our *in vitro* live imaging analysis of axonal transport dynamics of mitochondria revealed that E50K overexpression alters neither the anterograde nor retrograde transport dynamics of mitochondria or the lengths of transported mitochondria in RGCs *in vitro*. A previous study indicated that Bax is necessary for somatic degeneration in RGC death in glaucomatous neurodegeneration; however, Bax is not essential for axonal degeneration of RGCs^38^. Thus, based on previous and current findings of Bax activation, impairment of mitochondrial dynamics, mitochondrial degradation, and autophagosome and/or mitophagosome formation in RGC somas overexpressing E50K *in vitro*, we propose an important possibility that the E50K mutation may initiate Bax-mediated impairment of mitochondrial dynamics and degradation of mitochondria in RGC somas in RGC degeneration and contributes to a consequent induction of mitochondrial degradation and dysfunction in RGC axons. Future studies will be necessary to examine whether inhibition of Bax activation rescues mitochondrial dysfunction in E50K-mediated RGC degeneration.

Since aging and oxidative stress, important environmental factors of glaucoma and CNS diseases, contribute to mitochondrial dysfunction in experimental glaucoma^18^,^19^,^23^,^24^,^37^ and in patients with POAG^63^,^64^, we propose another possibility that E50K expression may have synergistic effects with aging and/or oxidative stress, and these effects may accelerate impairments of mitochondrial dynamics and function in the axons of age-related glaucomatous RGC degeneration. As strong support for this possibility, emerging evidence from our group has indicated that axonal transport of mitochondria is more vulnerable to glaucomatous insults in old mice than in young adult mice^44^. This finding suggests that a reduction in the capacity to resist stresses with aging, caused by decreased energy production and decreased support by mitochondria, may contribute to the age-related increase in glaucoma incidence^44^. Furthermore, mitochondrial oxidative stress caused by SOD2 deficiency promotes cellular senescence and aging phenotypes^65^, and *SOD2* gene transfer protects against optic nerve neuropathy induced by deficiency of OXPHOS Cx I 66. Importantly, it has been reported that E50K mutation is associated with oxidative stress and antioxidants reduced E50K-induced cell death and oligomer formation^49^,^50^. As discussed above, the E50K mutation alone does not affect mitochondrial transport and length in RGC axons *in vitro*, but it induces fission-mediated mitochondrial degradation and oxidative stress by increasing ROS production in RGC somas *in vitro*. Therefore, it is possible that aging and/or oxidative stress may act synergistically to induce mitochondrial degradation and dysfunction in the axons of glial lamina in aged E50K^−tg^ mice. At any rate, the significance of environmental risk factors such as aging and oxidative stress in E50K mutation-mediated neurodegeneration in POAG/NTG and ALS remains to be determined.

In summary, the present study connects for the first time mitochondrial pathogenic mechanisms to E50K mutation-induced RGC degeneration. E50K mutation activates the Bax pathway and oxidative stress and triggers dynamics-related mitochondrial degradation and mitophagy induction in RGC somas. E50K mutation however does not affect transport dynamics and fission of mitochondria in RGC axons. We propose that E50K mutation is involved in not only somatic mitochondrial degradation and mitophagy by alterations of Bax and/or mitochondrial dynamics in glaucomatous RGC degeneration but also axonal mitochondrial degradation and mitophagy in synergy with environmental risk factors such as aging and/or oxidative stress. Therefore, combinational therapeutic strategies that block susceptible target genes and environmental risk factors such as the effects of aging and oxidative stress, and protect both RGC somas and their axons in the pathogenesis of POAG are likely to be beneficial.

## Methods

### Animals

All procedures concerning animals were in accordance with the Association for Research in Vision and Ophthalmology (ARVO) for the use of animals in research and under protocols approved by Institutional Animal Care and Use Committee at the University of California, San Diego. The WT and E50K^−tg^ mice used in this study has been described previously^15^. To generate OPTN transgenic mice, mouse OPTN cDNA constructs were used in this study^15^. Female pregnant Sprague-Dawley rats (250–300 g in weight; Harlan Laboratories) were housed in covered cages, fed with a standard rodent diet ad libitum, and kept on a 12-h light/12-h dark cycle.

### Tissue preparation

Mice were anesthetized with intraperitoneal (IP) injection of a mixture of ketamine (100 mg/kg, Ketaset; Fort Dodge Animal Health) and xylazine (9 mg/kg, TranquiVed; VEDCO Inc.) before cervical dislocation. For immunohistochemistry, the retinas and ONHs were dissected from the choroids and fixed for 2 h at 4 °C with 4% paraformaldehyde (Sigma-Aldrich) in phosphate buffered saline (PBS, pH 7.4). After several washes in PBS, the retinas were dehydrated through graded ethanols and embedded in polyester wax. For immunoblot analyses, extracted retinas were immediately used.

### Primary RGC culture system

RGCs from postnatal 3–5 days of Sprague-Dawley rat were purified by immunopanning as described previously^67^. Briefly, approximately 15,000 purified cells were seeded on 24-well plates coated first with poly-D-lysine (PDL; 70 kDa, 10 μg/ml; Sigma-Aldrich) and then with laminin (10 μg/ml; Sigma-Aldrich) in neurobasal medium. RGCs were cultured in serum-free defined growth medium containing BDNF (50 μg/ml; PeproTech), CNTF (10 μg/ml; Sigma-Aldrich), insulin (5 μg/ml; Sigma-Aldrich), and forskolin (10 μg/ml; Sigma-Aldrich).

### *In vitro* transduction of recombinant AAV2 constructs

The AAV2-cytomegalovirus promoter (CMV)-pOPTN_WT_-GFP, pOPTN_E50K_-GFP, and GFP were produced using the pAAV-CMV-shuttle by Applied Viromics (Fremont). For production of recombinant AAV2 constructs, human OPTN cDNA constructs were used. For transduction of AAV2 constructs, purified RGCs were first mixed with AAV2 constructs (1 × 10^12^ GC/ml) and then plated on 12 mm coverslips in 24 well plates 35 mm Matek dishes or 60 mm dishes. Two days following transduction at 37 °C in a 10% CO_2_ incubator, cultured RGCs were fixed with 4% paraformaldehyde (Sigma-Aldrich) in PBS (pH 7.4) for light microscopy. For Western blot analyses, cultured RGCs were immediately used as described below.

### Immunoblot analysis

The retinal tissues or cultured primary RGCs were immediately homogenized in a glass-teflon Potter homogenizer in a modified RIPA lysis buffer (150 mM NaCl, 1 mM EDTA, 1% NP-40, 0.1% SDS, 1 mM DTT, 0.5% sodium deoxycholate and 50 mM Tris-Cl, pH 7.6), containing the complete protease inhibitors (Roche Biochemicals). Ten micrograms of pooled retinal protein (*n* = 4 retinas/group) and cultured RGC protein extracts from each group were separated by PAGE and electro-transferred to polyvinylidenedifluoride membranes. The membrane was blocked with 5% nonfat dry milk/0.5% Tween-20/phosphate buffered saline (PBS) for one hour and subsequently incubated with the primary antibodies overnight. The primary antibodies include mouse monoclonal anti-Bax antibody (1:1000; Santa Cruz Biotechnology), rabbit polyclonal anti-Bcl-xL antibody (1:1000; Cell Signaling), rabbit polyclonal anti-BDNF (1:1000; Santa Cruz Biotechnology), rabbit polyclonal anti-phospho-CREB S133 (1:1000; Millipore), mouse monoclonal anti-CypD antibody (1:1000; Life Technologies, Grand Island), mouse monoclonal anti-DRP1 antibody (1:1000, BD Transduction Laboratories), rabbit polyclonal anti-phospho-DRP1 S616 antibody (1:1000, Cell Signaling), rabbit polyclonal anti-LC3 antibody (1:3000; MBL International), mouse monoclonal OPTN antibody (1:1000; Santa Cruz Biotechnology), mouse monoclonal anti-total OXPHOS Cx antibody (containing a mixture of antibodies to CxI-IV and ATP synthase, 1:4000; Life Technologies), rabbit polyclonal anti-SOD2 antibody (1:3000; Santa Cruz Biotechnology), rabbit polyclonal anti-Tfam antibody (1:3000; Santa Cruz Biotechnology), and mouse monoclonal anti-actin antibody (1:10,000, Millipore) and the membrane was incubated with the primary antibodies for 16 h at 4 °C. After several washes in Tween/PBS (PBST), the membranes were incubated with peroxidase-conjugated goat anti-mouse IgG (1:5000; Bio-Rad) or goat anti-rabbit IgG (1:5000; Bio-Rad) for 2 h at room temperature and developed using chemiluminescence detection (ECL Plus; GE Healthcare Bio-Science). The blots were analysed by ImageQuant LAS 4000 and Image Quant TL 8.1 Software Package (GE Healthcare Bio-Sciences, Pittsburgh, PA). The band densities were normalized to the band densities for actin.

### Immunohistochemistry

Immunohistochemical staining of 7 μm wax sections of full thickness retina or ONH was performed. Primary antibodies included mouse monoclonal anti-GFAP antibody (1:500; Sigma-Aldrich), rabbit polyclonal anti-LC3 antibody (1:500; MBL International), and mouse monoclonal anti-NF68 (clone NR4, 1:500; Sigma-Aldrich). To prevent non-specific background, tissues were incubated in 1% bovine serum albumin/PBS for 1 h at room temperature before incubation with the primary antibodies for 16 h at 4 °C. After several wash steps, the tissues were incubated with the secondary antibody, Alexa Fluor 488 dye-conjugated goat anti-mouse or rabbit IgG (1:100, Life Technologies), for 4 h at 4 °C and subsequently washed with PBS. The sections were counterstained with the nucleic acid stain Hoechst 33342 (1 μg/mL; Life Technologies) in PBS. Images were acquired with confocal microscopy (Olympus FluoView1000; Olympus). ImageJ (http://rsb.info.nih.gov/ij/) was used to measure the fluorescence intensity in pixels per area in each NF68 and GFAP images from WT and E50K^−tg^ mice. In each antibody image acquisition, all imaging parameters remain the same. Mean pixel intensity was measured in this 262144 square pixel area (pixel size = 0.165 um).

### Transmission electron microscopy

For conventional EM, two eyes from each group (*n* = 2 mice) were fixed at 37 °C via cardiac perfusion with solution in 2% paraformaldehyde (Sigma-Aldrich) and 2.5% glutaraldehyde (Ted Pella) in 0.15M sodium cacodylate (pH 7.4, Sigma-Aldrich) and placed in pre-cooled fixative on ice for 1 h. The ONHs were dissected in 0.15M sodium cacodylate plus 3mM calcium chloride (pH 7.4) on ice and then post-fixed with 1% osmium tetroxide, 0.8% potassium ferrocyanide, 3 mM calcium chloride in 0.1M sodium cacodylate (pH 7.4) for 1 h, washed with ice-cold distilled water, poststained with 2% uranyl acetate at 4 °C, dehydrated using graded ethanols, and embedded in Durcupan resin (Fluka). Ultrathin (70 nm) sections were post-stained with uranyl acetate and lead salts and evaluated by a FEI spirit transmission EM operated at 120kV equipped with 2048 × 2048 pixel CCD camera. For quantitative analysis, the number of mitochondria was normalized to the total area occupied by axons in each image, which was measure using ImageJ (http://rsb.info.nih.gov/ij/). Mitochondrial lengths were measured with ImageJ (http://rsb.info.nih.gov/ij/). The mitochondrial volume density, defined as the volume occupied by mitochondria divided by the volume occupied by the axoplasm, was estimated using stereology. Briefly, a 112 × 112 square grid (112 × 112 chosen for ease of use with Photoshop) was overlaid on each image loaded in Photoshop (Adobe Systems Inc.), and mitochondria and axoplasm lying under intercepts were counted. The relative volume of mitochondria was expressed as the ratio of intercepts coinciding with this organelle relative to the intercepts coinciding with axoplasm.

### Electron microscope tomography

For each reconstruction, a double-tilt series of images at 1-degree tilt increments was collected with a FEI titan intermediate-voltage electron microscope operated at 300 kV and equipped with a 4096 × 4096 pixel CCD camera. The IMOD package was used for rough alignment with the fine alignment and reconstruction performed using the TxBR package. Volume segmentation was performed by manual tracing in the planes of highest resolution with IMOD. The mitochondrial reconstructions were visualized using IMOD and Amira. These programs allow one to step through slices of the reconstruction in any orientation and to track or model features of interest in three dimensions.

### Cell viability and cellular ATP measurements

Cell viability was measured in cultured RGCs transduced with AAV2-GFP, AAV2-OPTN_WT_-GFP or AAV2-OPTN_E50K_-GFP using MTT assay according to the manufacturer’s recommendations (Cell Proliferation Kit 1; Roche Diagnostics). In brief, cells were grown in 96-well plates with a final volume of 100 μl culture medium per well. Two days following transduction, 10 μl of the MTT labeling reagent (final concentration 0.5 mg/ml) was added to each well and the cultures were incubated in a conventional CO_2_ incubator at 37 °C for 4 h. Next, 100 μl of the solubilization solution was added into each well and the plates were incubated at 37 °C for 16 h in a humidified atmosphere of 10% CO_2_ incubator. Absorbance at 560 nm was then measured using a microplate reader (Spectra MAX; Molecular Devices Corp.). Data were presented as the percentage of cell viability in same day control wells, respectively. The level of cellular ATP in cultured RGCs transduced with AAV2-pOPTN_WT_-GFP or AAV2-pOPTN_E50K_-GFP was determined using a luciferase-based assay (CellTiter-Glo^TM^, Promega Corporation) according to the manufacturer’s recommendations). After the plates were developed, luminescence was measured in a microplate luminometer (Luminoskan, Labsystems). Each set of data was collected from multiple replicate wells of each experimental group (*n* = 6).

### ROS measurements

The intracellular ROS was measured by CellROX^®^ Deep Red Reagent (Life Technologies, New York, NY), useful as an indicator for ROS in cells. Briefly, RGCs transduced with AAV2-GFP or AAV2-OPTN_E50K_-GFP were plated on a 12-well plate (1 × 10^5^ cells/well) and cultured in a 10% CO_2_ incubator at 37 °C for 2 days. The cells were incubated with 100 nM CellROX^®^ Deep Red at 37 °C for 20 min and detached with Trypsin/EDTA. After washing with DPBS, the fluorescence of the sample was measured immediately using a flow cytometry (BD Accuri^TM^ C6, BD Bioscience, San Diego, CA). Each set of data was collected from multiple replicate dishes of each experimental group (n = 3).

### *In vitro* time-lapse imaging of RGCs

The purified RGCs were mixed with AAV2-GFP or AAV2-pOPTN_E50K_-GFP in the culture medium (300 μl), and plated at a density of 6000 cells/cm^2^ on 35-mm dishes coated with poly-D-lysine (Sigma-Aldrich) and laminin (Life Technologies). After 2 days in culture, mitochondria were stained by 12.5 nM MitoTracker Red CMXRos (Life Technologies) at 37 °C in 5% CO_2_ for 15 min for *in vitro* time-lapse imaging. Using a confocal laser-scanning inverted microscope (FluoView FV10i; Olympus) equipped with a chamber kept at 37 °C and 5% CO_2_, we first identified GFP^+^ RGCs and then *in vitro* time-lapse imaging (every 3 s for 3 min) of axonal transport of mitochondria in the GFP-positive RGCs was conducted using a 60× water-immersion objective with a numerical aperture of 1.2 (Olympus).

### Quantification of axonal transport and length of mitochondria in RGCs *in vitro*

The images of RGCs were analyzed using MetaMorph software (Molecular Devices) and the indicated modules. Kymographs were generated using Stack Arithmetic (maximum) and by drawing a region of interest. In kymographs, diagonal lines represent mitochondrial movement, whereas vertical lines represent stationary mitochondria and/or varicosities. We defined axonal transport of mitochondria as mitochondrial movement with a velocity of ≥0.05 μm/s. Diagonal lines of velocity < 0.05 μm/s were regarded as pause. The duty cycle was calculated as the percentage of time spent in transport. The velocity of axonal transport of mitochondria was calculated as the distance of transport without pauses divided by the duration of transport. Mitochondria-free regions in axons were measured using the kymograph from the first to the second plane by the Integrated Morphometry Analysis function. The length of mitochondria was defined as the length parallel to the axonal long axis and only one segment per axon for quantification of axonal transport of mitochondria was analyzed.

### Statistical analysis

Data were presented as either mean ± SD or mean ± SEM. Comparison of two or three experimental conditions was evaluated using unpaired, two-tailed Student’s *t*-test, or one-way analysis of variance and the Bonferroni *t*-test. When normality was questioned, the Mann-Whitney test was used. *P* < 0.05 was considered to be statistically significant.

## Additional Information

**How to cite this article**: Shim, M. S. *et al*. Mitochondrial pathogenic mechanism and degradation in optineurin E50K mutation-mediated retinal ganglion cell degeneration. *Sci. Rep.* **6**, 33830; doi: 10.1038/srep33830 (2016).

## Supplementary Material

Supplementary Movie 1

Supplementary Movie 2

Supplementary Movie 3

Supplementary Information
