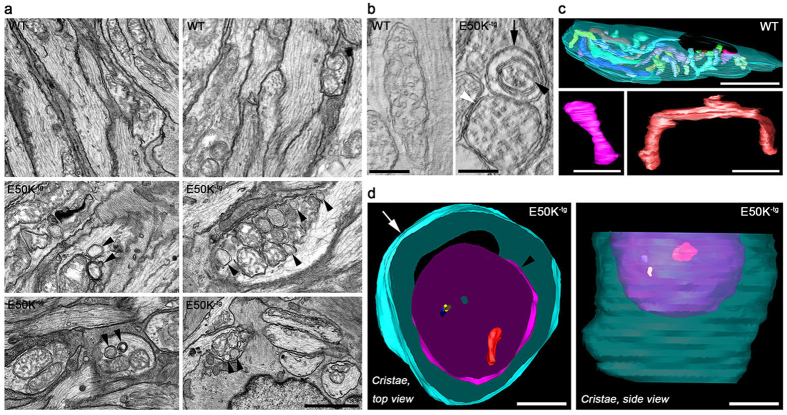# Corrigendum: Mitochondrial pathogenic mechanism and degradation in optineurin E50K mutation-mediated retinal ganglion cell degeneration

**DOI:** 10.1038/srep40460

**Published:** 2017-01-19

**Authors:** Myoung Sup Shim, Yuji Takihara, Keun-Young Kim, Takeshi Iwata, Beatrice Y. J. T. Yue, Masaru Inatani, Robert N. Weinreb, Guy A. Perkins, Won-Kyu Ju

Scientific Reports 6: Article number: 33830; 10.1038/srep33830 published online: 09 22 2016; updated: 01 19 2017.

This Article contains an error in panel C of Figure 4, where “WT” was incorrectly written as “E50K^−tg^”. The correct Figure 4 appears below as Figure 1.

## Figures and Tables

**Figure 1:**