# Supplementary material for: Mitochondrial pathogenic mechanism and degradation in optineurin E50K mutation-mediated retinal ganglion cell degeneration
Source: Sci Rep. 2016 Sep 22;6:33830. doi: 10.1038/srep33830 (PMC5031982; doi:10.1038/srep33830)

Supplementary Information

Mitochondrial pathogenic mechanism and degradation in optineurin E50K mutation-mediated retinal ganglion cell degeneration

Myoung Sup Shim, Yuji Takihara, Keun-Young Kim, Takeshi Iwata, Beatrice Y.J.T. Yue, Masaru Inatani, Robert N. Weinreb, Guy A. Perkins, and Won-Kyu Ju

Supplementary Materials and Methods

Animals. All procedures concerning animals were in accordance with the Association for Research in Vision and Ophthalmology (ARVO) for the use of animals in research and under protocols approved by Institutional Animal Care and Use Committee at the University of California, San Diego. Adult female DBA/2J and DBA/2J-*Gpnmb*$^{+}$ (D2-*Gpnmb*$^{+}$) mice (The Jackson Laboratory), and female pregnant Sprague-Dawley rats (250 - 300 g in weight; Harlan Laboratories) were housed in covered cages, fed with a standard rodent diet ad libitum, and kept on a 12-h light/12-h dark cycle.

IOP measurement. IOP elevation onset typically occurs between 5 and 7 months of age, and by 9 to 10 months of age, IOP-linked optic nerve axon loss is well advanced[1-4]. IOP measurements were performed as previously described[4,5]. Each of the 9-mo-old DBA/2J mice used in this study had a single IOP measurement (to confirm development of spontaneous IOP elevation exceeding 20 mmHg) (n = 10 for DBA/2J mice). Also, each of the non-glaucomatous control D2-*Gpnmb*$^{+}$ mice (n = 10) used in this study had a single IOP measurement. After anesthesia with intraperitoneal [IP] injection of a mixture of ketamine (100 mg/kg, Ketaset; Fort Dodge Animal Health, Fort Dodge, IA) and xylazine (9 mg/kg, TranquiVed, Vedeco, Inc.), a sterilized, water-filled microneedle with an external diameter of 50 to 70 µm was used to cannulate the anterior chamber. The microneedle was then repositioned to minimize corneal deformation and to ensure that the eye remained in its normal position. The microneedle was connected to a pressure transducer (Blood Pressure Transducer; WPI), which relayed its signal to a bridge amplifier (Quad Bridge; AD Instruments [ADI]). The amplifier was connected to an analog-to-digital converter (Power Laboratory; ADI) and a computer (G4 Macintosh; Apple Computer Inc.). After measuring the IOP, we used the glaucomatous DBA/2J mice that had spontaneous IOP elevation exceeding 20 mmHg and the mice were confirmed by examining the ON axon damage[3,4].

***In vitro* transduction of recombinant AAV2 constructs.** The AAV2-cytomegalovirus promoter (CMV)-pOPTN$_{E50K}$-GFP, pOPTN$_{WT}$-GFP and GFP were produced using the pAAV-CMV-shuttle by Applied Viromics (Fremont). For transduction of AAV2 constructs, purified RGCs were first mixed with AAV2 constructs (1 X 10^{12} GC/ml) and then plated on 35 mm Matek dishes (Ted Pella). Following 2 days after transduction in a 10% CO_2 incubator, protein extracts from cultured RGCs were immediately used for immunoblot analysis.

Immunoblot analysis. The retinal tissues and cultured RGCs were then immediately homogenized in a glass-teflon Potter homogenizer in lysis buffer (20mM Hepes, pH 7.5/10mM KCl/1.5mM $MgCl_2$/1mM EDTA/1mM DTT/0.5% CHAPS/complete protease inhibitors; Roche

Biochemicals). Ten micrograms of pooled retinal protein (*n* = 4 retinas/group) and cultured RGCs from each group were separated by PAGE and electro-transferred to polyvinylidenedifluoride membranes. The membrane was blocked with 5% nonfat dry milk/0.5% Tween-20/PBS for one hour and subsequently incubated with the primary antibodies overnight. The primary antibodies included rabbit polyclonal anti-BDNF (1:1000; Santa Cruz Biotechnology), mouse monoclonal anti-Bax antibody (1:1000; Santa Cruz Biotechnology), rabbit polyclonal anti-pCREB S133 (1:1000; Millipore), mouse monoclonal OPTN antibody (1:1000; Santa Cruz Biotechnology) and mouse monoclonal anti-actin antibody (1:10,000, Millipore) and the membrane was incubated with the primary antibodies for 16 h at 4°C. After several washes in Tween/PBS (PBST), the membranes were incubated with peroxidase-conjugated goat anti-mouse IgG (1:5000; Bio-Rad) or goat anti-rabbit IgG (1:5000; Bio-Rad) for 2 h at room temperature. After several washes in Tween/PBS (PBST), the membranes were incubated with peroxidase-conjugated goat anti-mouse IgG (1:5000; Bio-Rad) or goat anti-rabbit IgG (1:5000; Bio-Rad) and developed using chemiluminescence detection (ECL Plus; GE Healthcare Bio-Science, Piscataway). The blots were analysed by ImageQuant LAS 4000 and Image Quant TL 8.1 Software Package (GE Healthcare Bio-Sciences, Pittsburgh, PA). The band densities were normalized to the band densities for actin.

Immunohistochemistry. Immunohistochemical staining of 7 μm wax sections of full thickness retina or ONH was performed. Primary antibodies included rabbit polyclonal anti-BDNF (1:200; Santa Cruz Biotechnology), mouse monoclonal anti-GFAP antibody (1:500; Sigma-Aldrich) and mouse monoclonal OPTN antibody (1:1000; Santa Cruz Biotechnology). To prevent non-specific background, tissues were incubated in 1% bovine serum albumin/PBS for 1 h at room temperature before incubation with the primary antibodies for 16 h at 4°C. After several wash steps, the tissues were incubated with the secondary antibody, Alexa Fluor 488 dye-conjugated goat anti-mouse IgG (1:100, Life Technologies) or Alexa Fluor 568 dye-conjugated goat anti-rabbit IgG (1:100, Life Technologies), for 4 h at 4°C and subsequently washed with PBS. The sections were counterstained with the nucleic acid stain Hoechst 33342 (1 μg/mL; Life Technologies) in PBS. Images were acquired with confocal microscopy (Olympus FluoView1000; Olympus)

Whole-mount immunohistochemistry

Retinas from enucleated eyes were dissected as flattened whole-mounts from 10-month glaucomatous DBA/2J mice. Retinas were immersed in PBS containing 30% sucrose for 24 h at 4°C. The retinas were blocked in PBS containing 3% donkey serum, 1% bovine serum albumin, 1% fish gelatin and 0.1% triton X-100, and incubated with goat polyclonal anti-Brn3a antibody (1:500, Santa Cruz Biotechnology) for 3 d at 4°C. After several wash steps, the retinas were incubated with the secondary antibody, Alexa Fluor-568 donkey anti-goat IgG antibody (1:100, Invitrogen), for 24 h, and subsequently washed with PBS. Images were captured with a spinning-disc confocal microscope (Olympus America Inc.).

Supplementary References

1. John S.W., Hagaman, J.R., MacTaggart, T.E., Peng, L. & Smithes, O. Intraocular pressure in inbred mouse strains. *Investigative ophthalmology & visual science* **38**, 249-253 (1997).

2. Libby, R.T. *et al.* Susceptibility to neurodegeneration in a glaucoma is modified by Bax gene dosage. *PLoS Genet* **1**, 17-26 (2005).

3. Howell, G.R. *et al.* Axons of retinal ganglion cells are insulted in the optic nerve early in DBA/2J glaucoma. *The Journal of cell biology* 179, 1523-1537 (2007).

4. Ju, W.K. *et al.* Intraocular pressure elevation induces mitochondrial fission and triggers OPA1 release in glaucomatous optic nerve. *Investigative ophthalmology & visual science* **49**, 4903-4911 (2008).

5. Aihara, M., Lindsey, J.D., Weinreb, R.N. Reduction of intraocular pressure in mouse eyes treated with latanoprost. *Investigative ophthalmology & visual science* **43**, 146-150 (2002).

Supplementary Figure Legends

Supplementary Figure 1. Reduction of BDNF expression in the retina of aged E50K^{-tg} mice. (**a**) Immunoblot analysis of BDNF and phospho-CREB (S133) protein in retinal extracts from 16-month-old aged E50K^{-tg} mice. For each determination, the actin level in WT was normalized to a value of 1.0. Data are shown as the mean ± S.D. (*n* = 3 independent experiments). ***P*<0.01 compared with the WT group. Full-length blots are presented in Supplementary Figure 10. (**b**) Immunohistochemical analysis of BDNF and GFAP protein expression in retinal sections from WT and E50K^{-tg} mice. BDNF immunoreactivity was highly decreased in RGCs of the GCL, however, GFAP immunoreactivity was induced in the müller cells in the inner retinal layer of E50K^{-tg} mice. BDNF, brain-derived neurotrophic factor; CREB, cyclic AMP response element-binding protein; E50K^{-tg}, E50K mutation-carrying transgenic; GCL, ganglion cell layer; GFAP, glial fibrillary acidic protein; IPL, inner plexiform layer; RGC, retinal ganglion cell; S133, serine 133; WT, wild-type. Scale bar, 20 μm.

Supplementary Figure 2. Enhanced expression of E50K triggers BDNF loss in cultured RGCs. Primary RGCs were transduced with AAV2-GFP or AAV2-OPTN$_{E50K}$-GFP for 2 days. Immunoblot analysis of BDNF and phospho-CREB (S133) protein in cultured RGCs overexpressing E50K mutant were performed. For each determination, the actin level in the control was normalized to a value of 1.0. Data are shown as the mean ± S.D. (*n* = 3 independent experiments). **P*<0.05 compared with the control group. Full-length blots are presented in Supplementary Figure 11. BDNF, brain-derived neurotrophic factor; CREB, cyclic AMP response element-binding protein; GFP, green fluorescent protein; RGC, retinal ganglion cell; S133, serine 133; WT, wild-type.

Supplementary Figure 3. IOP elevation and RGC loss in glaucomatous DBA/2J mice. (a) The representative actual IOP values (*n* = 10 mice per group). (b) The average IOP. Data are shown as the mean ± S.D. (c) RGC loss in 10-month-old glaucomatous DBA/2J mice. Representative images show retinal whole-mounts from non-glaucomatous control D2-*Gpnmb*$^{+}$ and glaucomatous DBA/2J mice. High magnification shows loss of RGC in glaucomatous DBA/2J mice compared with D2-*Gpnmb*$^{+}$ mice. D2, DBA/2J; IOP, intraocular pressure. Scale bar, 100 μm.

Supplementary Figure 4. Reduction of OPTN protein expression in the retinal extracts of glaucomatous DBA/2J mice. (**a**) Immunoblot analysis of OPTN and Bax proteins in retinal extracts from 10-month-old DBA/2J mice. For each determination, the actin level in WT was normalized to a value of 1.0. Data are shown as the mean ± S.D. (*n* = 3 independent experiments). ***P*<0.05 compared with D2-*Gpnmb*$^{+}$ group. Full-length blots are presented in Supplementary Figure 12. (**b**) Immunohistochemical analysis of OPTN protein expression in the retinal section from D2-*Gpnmb*$^{+}$ and DBA/2J mice. OPTN immunoreactivity was highly decreased in RGCs of the GCL and the inner retinal layers of DBA/2J mice. D2, DBA/2J; INL, inner nuclear layer; IPL, inner plexiform layer; GCL, ganglion cell layer; IPL, inner plexiform layer; ONL, outer nuclear layer; OPL, outer plexiform layer; OPTN, optineurin; RGC, retinal ganglion cell. Scale bar, 20 μm.

Supplementary Figure 5. Original blots for Figure 1.

Supplementary Figure 6. Original blots for Figure 2.

Supplementary Figure 7. Original blots for Figure 5.

Supplementary Figure 8. Original blots for Figure 6.

Supplementary Figure 9. Original blots for Figure 7.

Supplementary Figure 10. Original blots for Supplementary Figure 1.

Supplementary Figure 11. Original blots for Supplementary Figure 2.

Supplementary Figure 12. Original blots for Supplementary Figure 4.

Supplementary Movie Legends

Supplementary Movie 1. 3D reconstruction of volume segmentation of the mitophagosome formation and aggregated cristae structure in cultured RGCs overexpressing E50K mutant.

Supplementary Movie 2. *In vitro* time-lapse imaging (every 3 s for 3 min) shows highly dynamic axonal transport of mitochondria in cultured RGCs transduced with AAV2-GFP. Time is indicated as min:s.

Supplementary Movie 3. *In vitro* time-lapse imaging (every 3 s for 3 min) shows highly dynamic axonal transport of mitochondria in cultured RGCs transduced with AAV2-$OPTN_{E50K}$-GFP. Time is indicated as min:s.

Supplementary Figure 1 and b

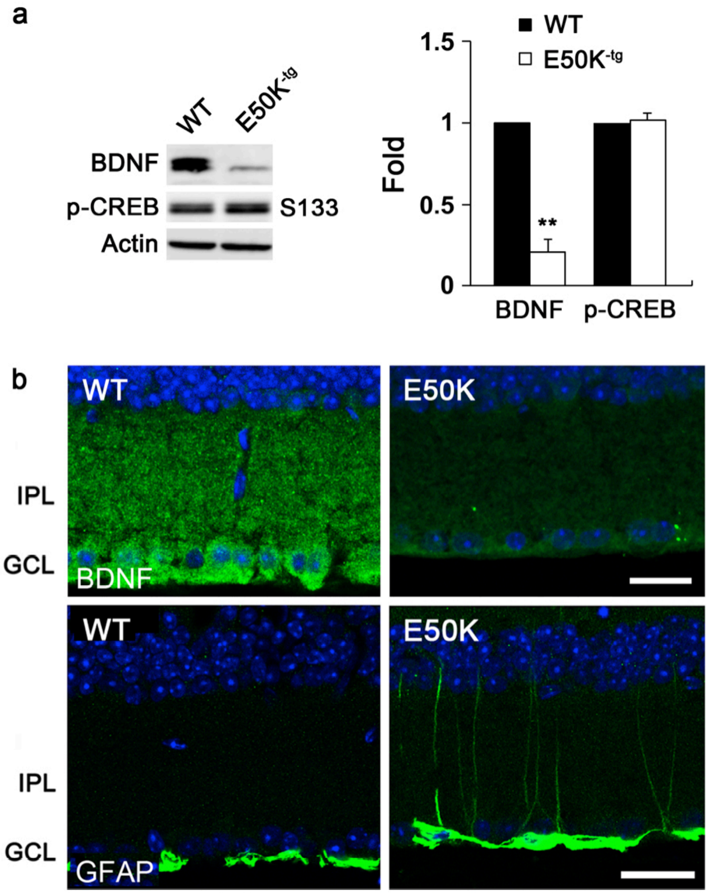

Supplementary Figure 2.

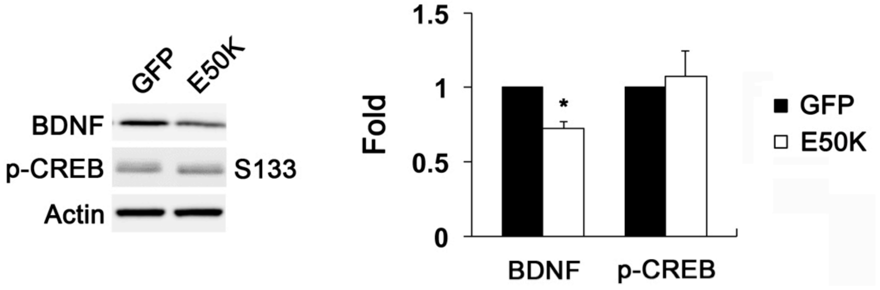

Supplementary Figure 3a-c.

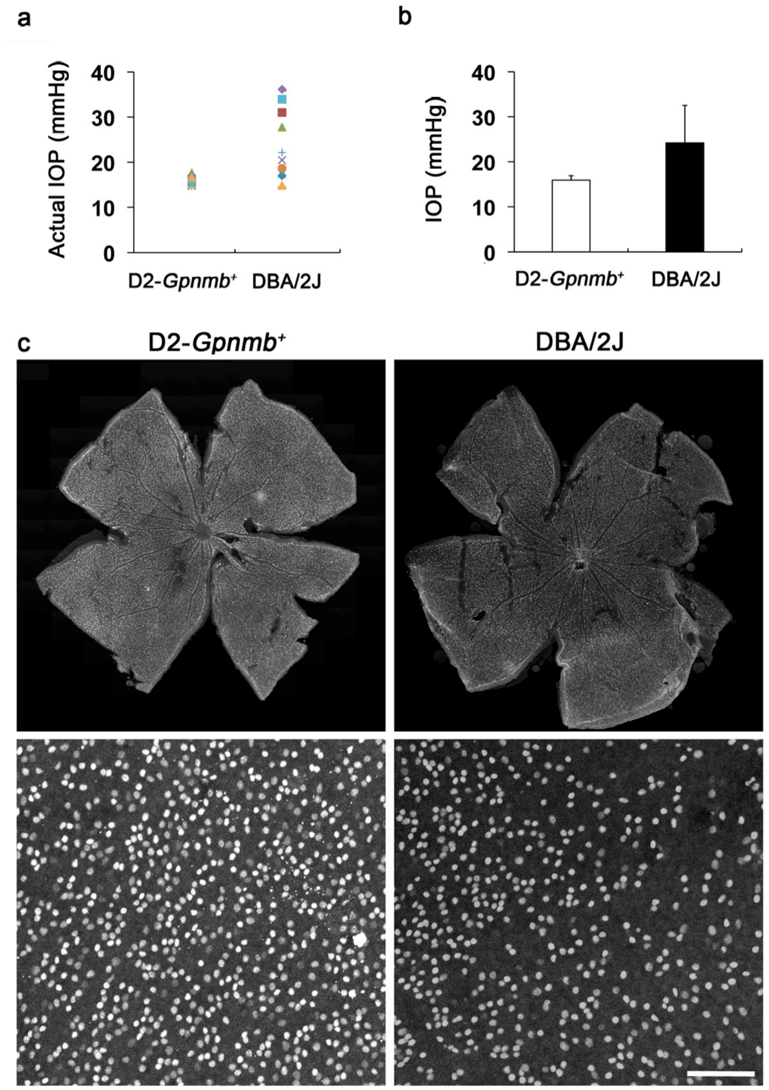

Supplementary Figure 4a and b.

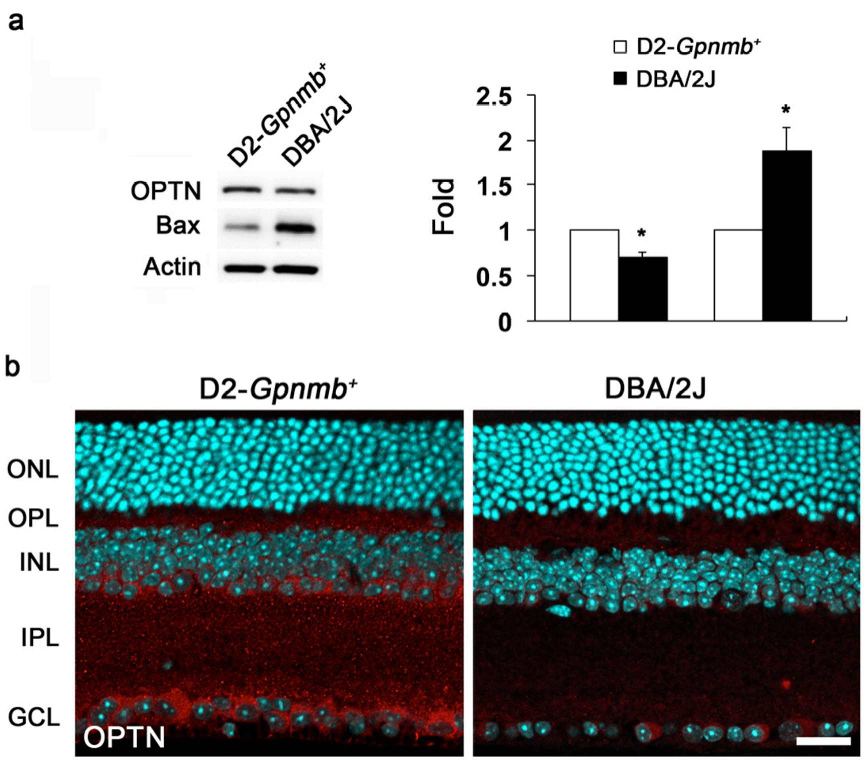

Supplementary Figure 5.

M: PageRuler
1: WT retina
2: E50K OPTN retina

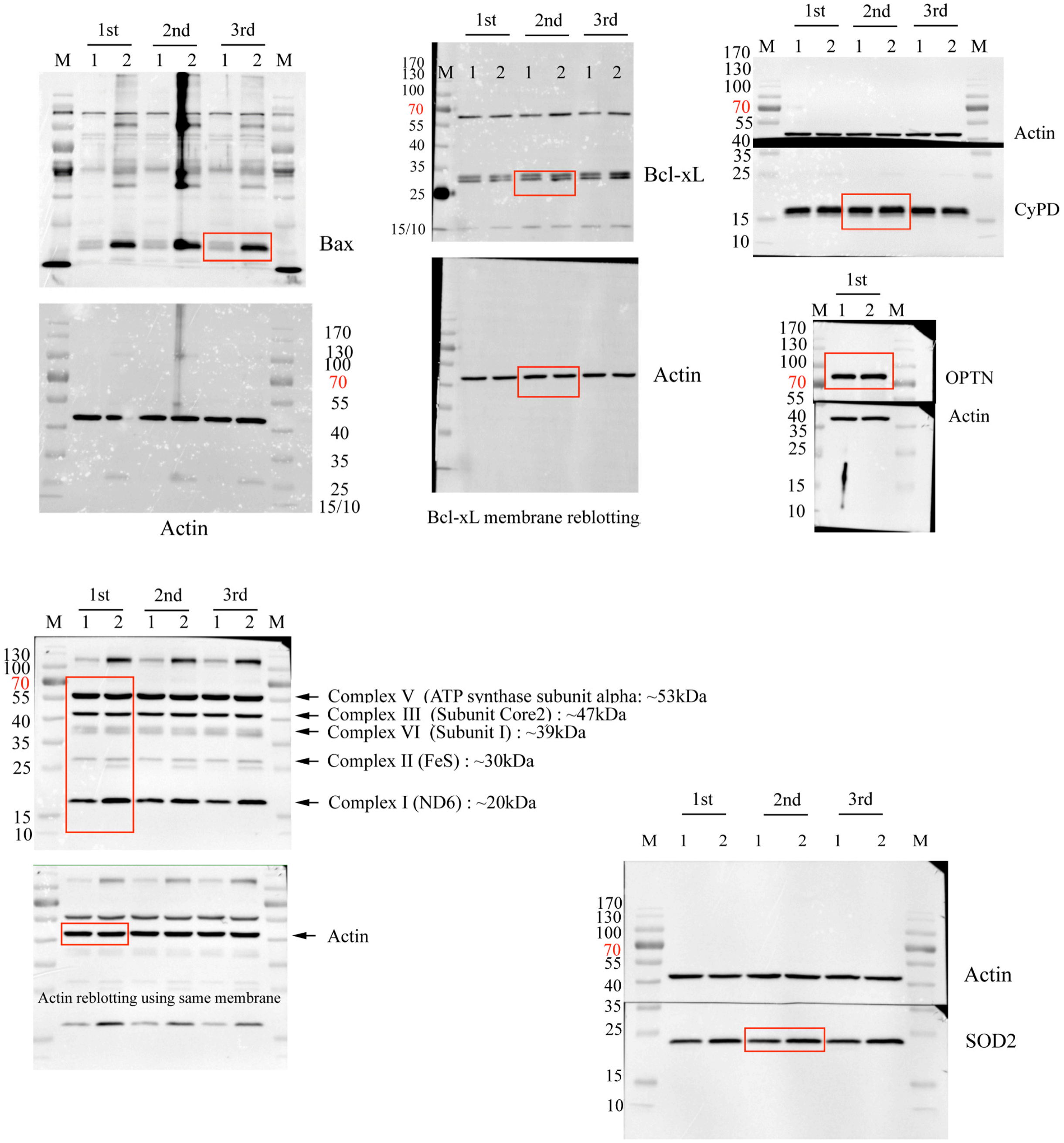

Supplementary Figure 6.

M: PageRuler
1: WT retina
2: E50K OPTN retina

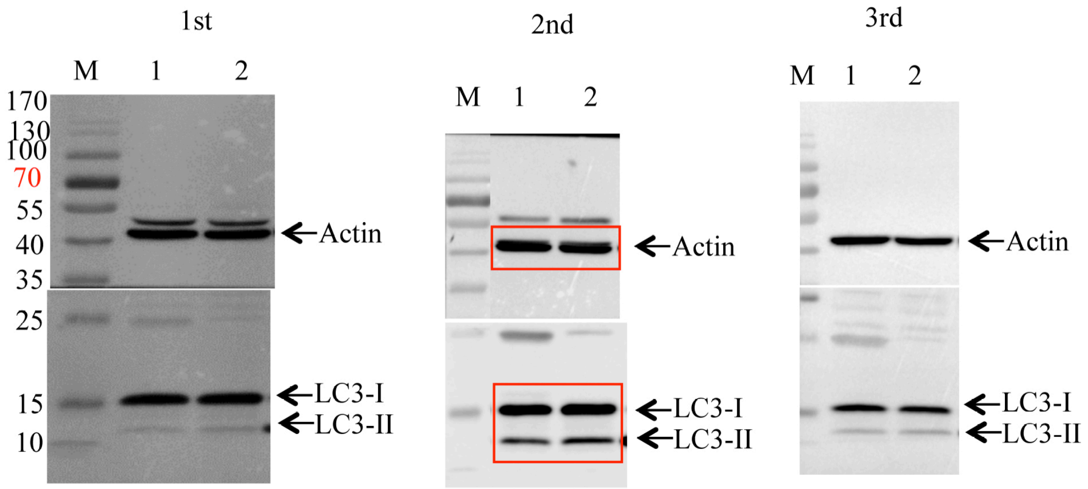

Supplementary Figure 7.

M: PageRuler
1: pRGC-GFP
2: pRGC-E50K-GFP

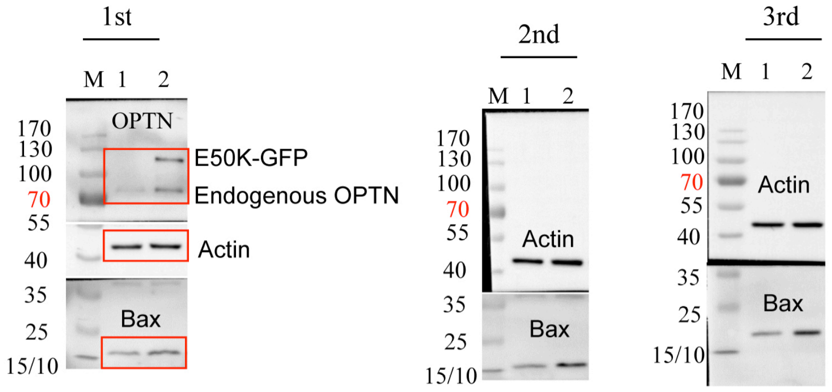

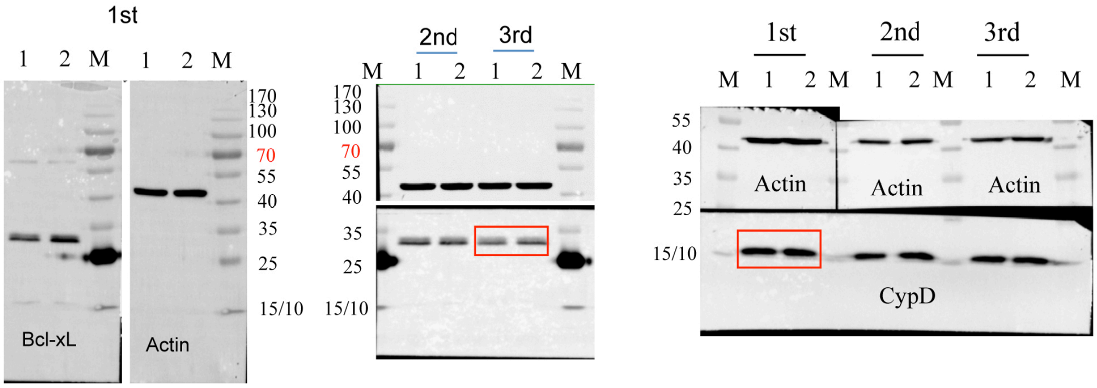

M: PageRuler
1: pRGC-GFP
2: pRGC-WT OPTN-GFP

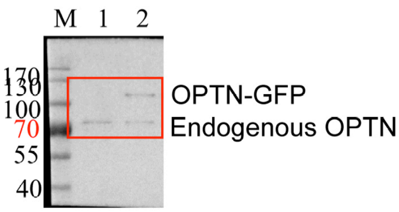

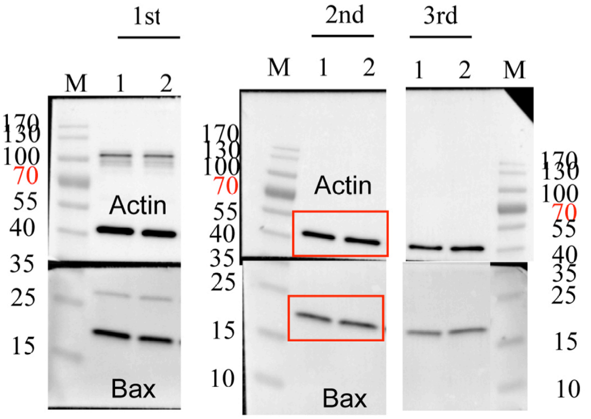

Supplementary Figure 8.

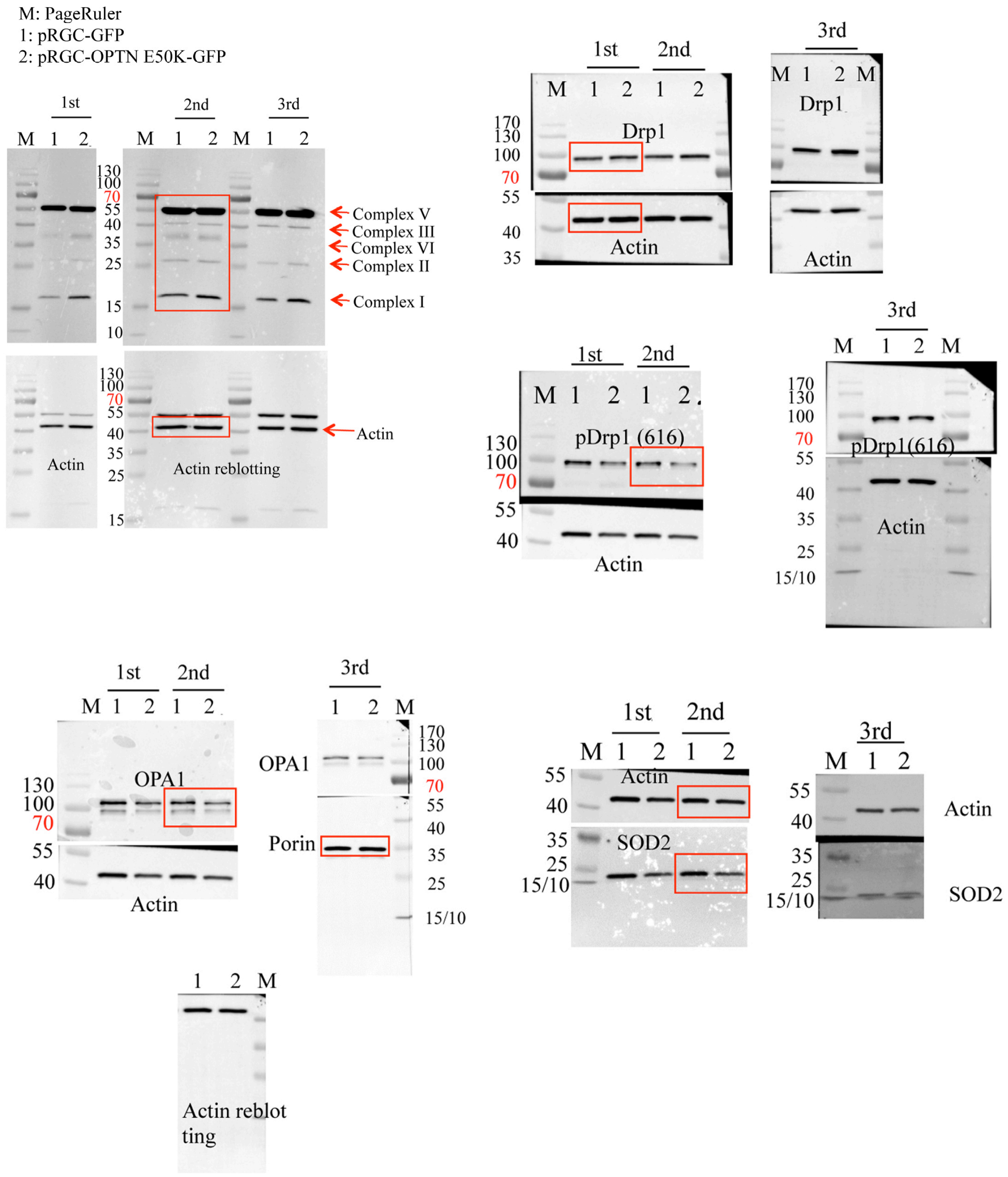

Supplementary Figure 9.

M: PageRuler
1: pRGC-GFP
2: pRGC-OPTN E50K-GFP

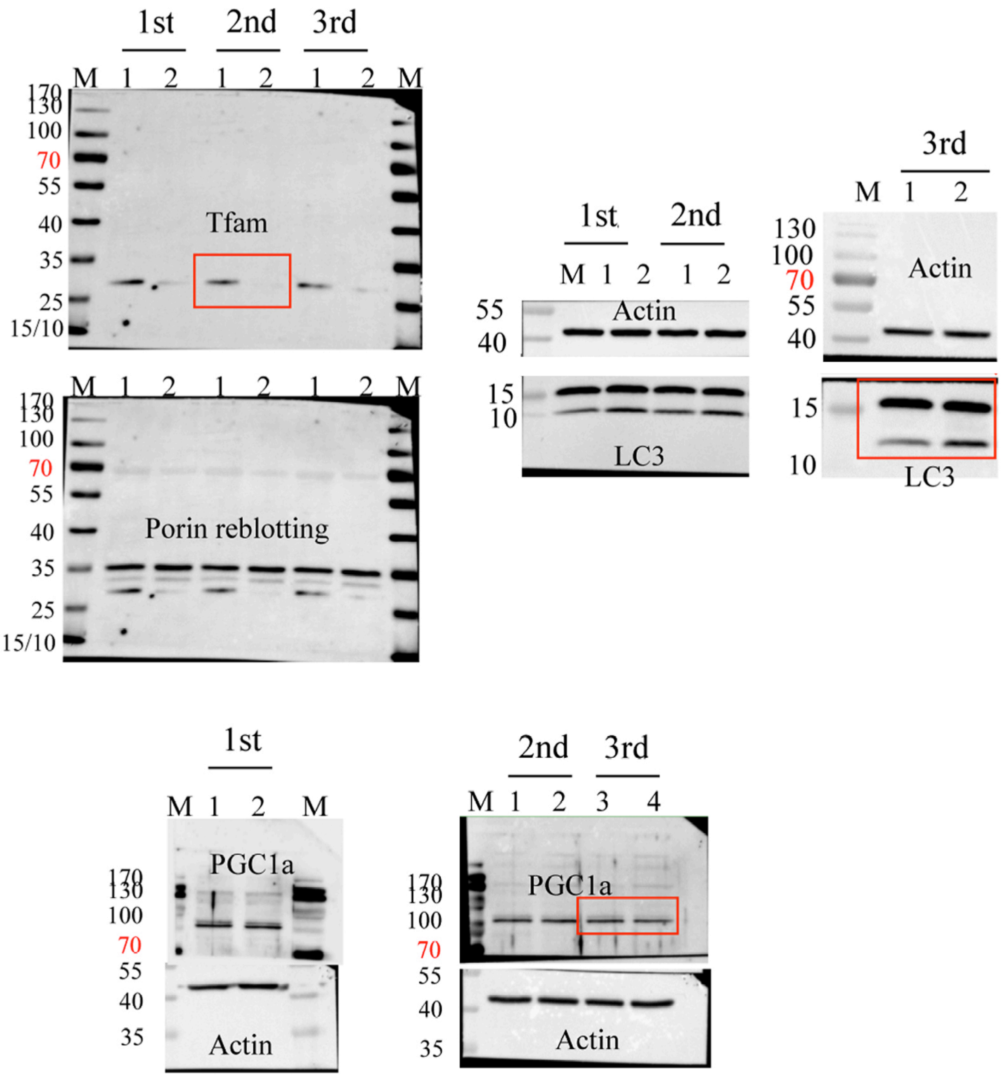

Supplementary Figure 10.

M: PageRuler
1: WT retina
2: E50K OPTN retina

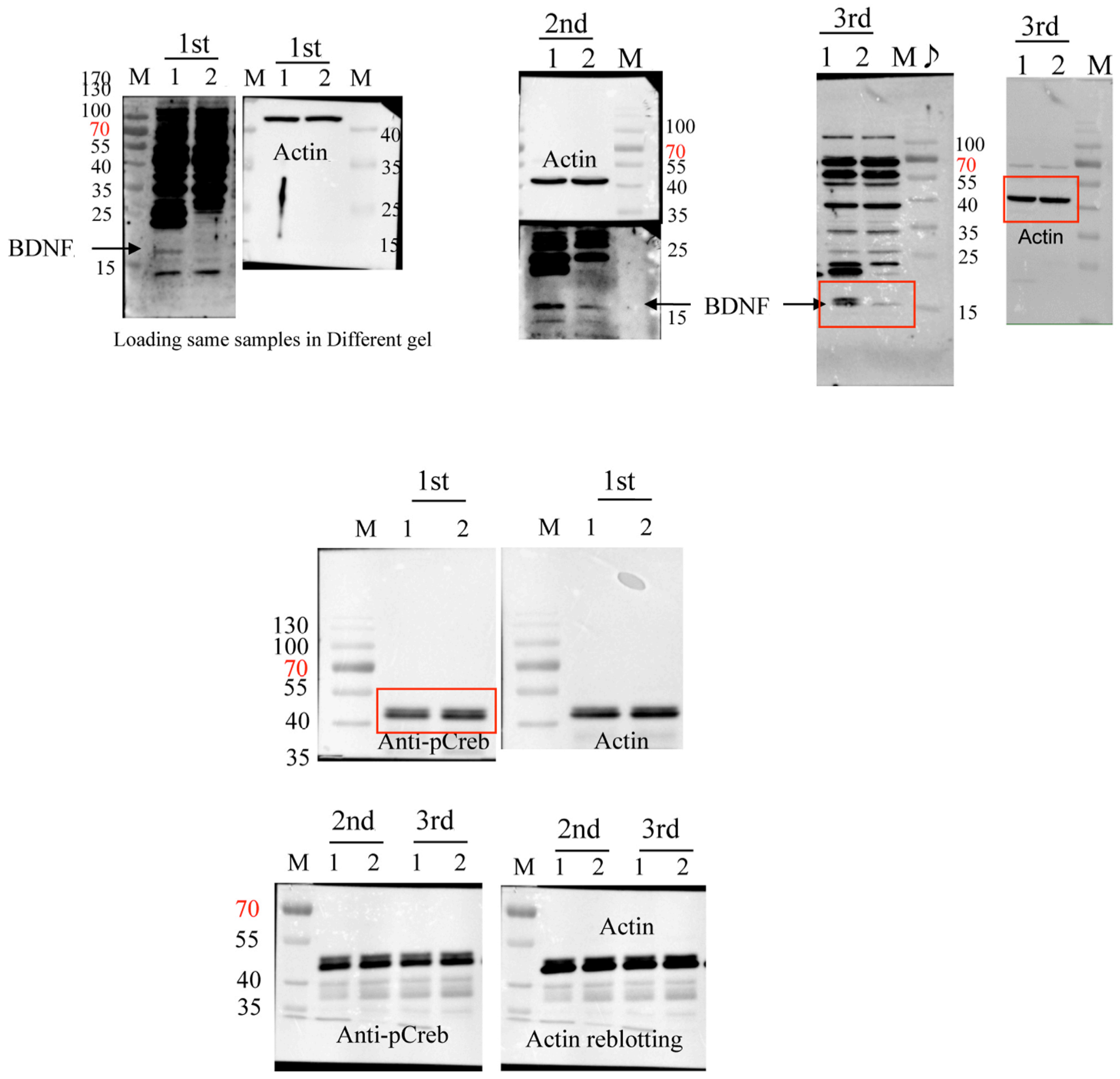

Supplementary Figure 11.

M: PageRuler
1: pRGC-GFP
2: pRGC-GFP-E50K

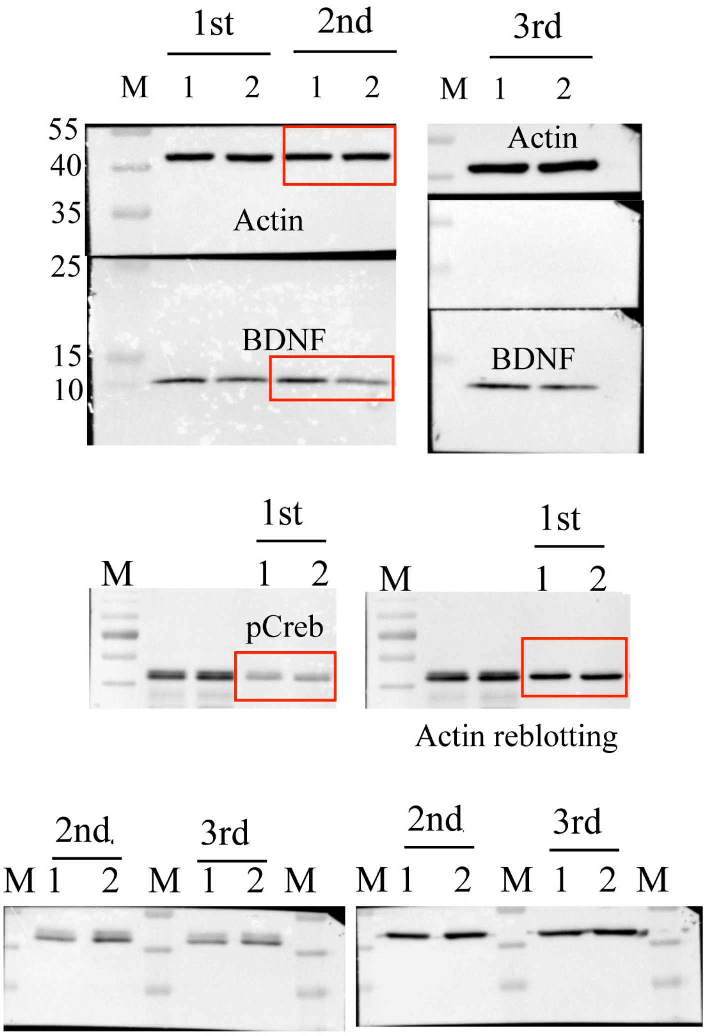

Supplementary Figure 12.

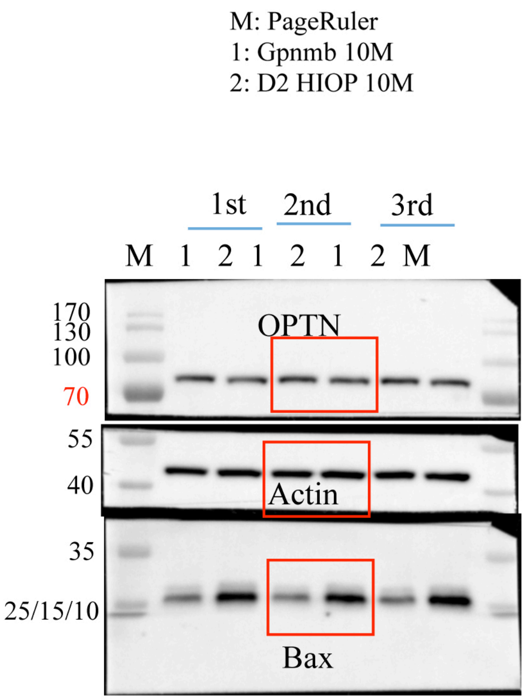

Supplement: Supplementary Information [file srep33830-s4.pdf]
